# CYLD induces high oxidative stress and DNA damage through class I HDACs to promote radiosensitivity in nasopharyngeal carcinoma

**DOI:** 10.1038/s41419-024-06419-w

**Published:** 2024-01-29

**Authors:** Yueshuo Li, Chenxing Yang, Longlong Xie, Feng Shi, Min Tang, Xiangjian Luo, Na Liu, Xudong Hu, Yongwei Zhu, Ann M. Bode, Qiang Gao, Jian Zhou, Jia Fan, Xuejun Li, Ya Cao

**Affiliations:** 1 Key Laboratory of Carcinogenesis and Cancer Invasion, Chinese Ministry of Education, Department of Neurosurgery, Xiangya Hospital, Central South University, Changsha, 410078 China; 2 Department of Neurosurgery, National Clinical Research Center for Geriatric Disorders/ Xiangya Hospital, Central South University, Changsha, 410078 China; 3 Key Laboratory of Carcinogenesis of National Health Commission, Cancer Research Institute and School of Basic Medical Science, Xiangya School of Medicine, Central South University, Changsha, 410078 China; 4 Hunan International Scientific and Technological Cooperation Base of Brain Tumor Research, Xiangya Hospital, Central South University, Changsha, Hunan 410008 China; 5 Children’s Hospital, Xiangya School of Medicine, Central South University, Changsha, Hunan 410008 China; 6 Molecular Imaging Research Center of Central South University, Changsha, 410008 Hunan China; 7 The Hormel Institute, University of Minnesota, Austin, MN 55912 USA; 8 Key Laboratory for Carcinogenesis and Cancer Invasion, Chinese Ministry of Education, Zhongshan Hospital, Shanghai Medical School, Fudan University, Shanghai, 200000 China; 9 Department of Radiology, National Clinical Research Center for Geriatric Disorders/ Xiangya Hospital, Central South University, Changsha, 410078 China; 10 Research Center for Technologies of Nucleic Acid-Based Diagnostics and Therapeutics Hunan Province, Changsha, 410078 China; 11 National Joint Engineering Research Center for Genetic Diagnostics of Infectious Diseases and Cancer, Changsha, 410078 China

**Keywords:** Cell signalling, Cancer therapy, DNA damage and repair

## Abstract

Abnormal expression of Cylindromatosis (CYLD), a tumor suppressor molecule, plays an important role in tumor development and treatment. In this work, we found that CYLD binds to class I histone deacetylases (HDAC1 and HDAC2) through its N-terminal domain and inhibits HDAC1 activity. RNA sequencing showed that CYLD-HDAC axis regulates cellular antioxidant response via Nrf2 and its target genes. Then we revealed a mechanism that class I HDACs mediate redox abnormalities in CYLD low-expressing tumors. HDACs are central players in the DNA damage signaling. We further confirmed that CYLD regulates radiation-induced DNA damage and repair response through inhibiting class I HDACs. Furthermore, CYLD mediates nasopharyngeal carcinoma cell radiosensitivity through class I HDACs. Thus, we identified the function of the CYLD-HDAC axis in radiotherapy and blocking HDACs by Chidamide can increase the sensitivity of cancer cells and tumors to radiation therapy both in vitro and in vivo. In addition, ChIP and luciferase reporter assays revealed that CYLD could be transcriptionally regulated by zinc finger protein 202 (ZNF202). Our findings offer novel insight into the function of CYLD in tumor and uncover important roles for CYLD-HDAC axis in radiosensitivity, which provide new molecular target and therapeutic strategy for tumor radiotherapy.

## Introduction

Abnormal expression of Cylindromatosis (CYLD) has an important role in tumor development. The CYLD structure contains an N-terminal and a C-terminal. The N-terminal is responsible for binding target molecules. The C-terminal contains a homologous ubiquitin-specific peptidase (USP) catalytic domain of the ubiquitin-specific protease family, which mediates the cleavage of the ubiquitin chain and is mainly responsible for protein function and stability [1–3]. Recently, we reported that CYLD stabilizes its protein level by binding to and removing the ubiquitin chain of the cell cycle inhibitory molecule p18, thereby negatively regulating cell cycle progression [4]. Decreased expression and enzymatic activity are the main features of CYLD in tumors [5–8]. Down-regulation of CYLD expression enhances the resistance to chemotherapy in breast cancer and hepatocellular carcinoma (HCC) [9, 10]. In addition, loss of CYLD expression causes cisplatin resistance in oral squamous cell carcinoma (OSCC) patients with NF-κB hyperactivation [11]. This suggests that CYLD could be considered a potential predictive biomarker and target for therapeutic resistance in various cancers.

Histone acetylation is regulated by the concerted actions of histone acetyltransferases (HATs) and histone deacetylases (HDACs) that work by adding and removing acetyl groups from lysine residues. Abnormal expression and activity of HDACs are high-frequency events in the process of tumor development. The classical HDAC family (Class I, II, IV) is involved in a variety of tumor types. Class I HDACs include HDAC1, HDAC2, HDAC3 and HDAC8. These HDACs are ubiquitously expressed and are mainly localized within the nucleus, where they function in diverse processes, including transcription. Importantly, class I HDACs, especially HDAC1 and HDAC2 are highly homologous and exist as a homo or heterodimer. Class I HDACs are also found to associated with tumor progression and poor prognosis [12, 13].

Nasopharyngeal carcinoma (NPC) is an important subtype of head and neck squamous cell carcinoma (HNSCC), which is strongly driven by Epstein–Barr virus [14, 15]. Radiotherapy is the main treatment strategy for NPC. Furthermore, radiotherapy is widely used for more than 50% of cancer patients, which utilizes high doses of radiation to destroy or delay the growth of tumors [16]. Radiotherapy causes DNA damage directly by ionization or indirectly via the generation of free radicals. Much of the damage from radiation is indirect, especially reactive oxygen species (ROS) that are generated by radiolysis of water. In response to excessive ROS, tumor cells increase their antioxidant capacity to establish a new redox state [17, 18]. Furthermore, studies have shown that Nrf2 activation and expression up-regulation mediates malignant phenotypic and radiotherapy resistance in tumor cells [19–21]. During radiation treatment, cancer cells evolve personalized regulation mechanisms against the insults of radiation in order to survive. Thus, radiation resistance to genotoxic therapy, as well as the nature of tumor heterogeneity, greatly limits the efficacy of cancer therapy resulting in treatment failure [22, 23]. Radiotherapy resistance is multimodal, involving multiple biological changes in the tumor itself and its tumor microenvironment, including cell cycle, hypoxia, oxidative stress, apoptosis, DNA damage repair, inflammation, and mitochondrial function [24]. Therefore, studying the molecular mechanisms of tumor radiotherapy, discovering new markers of radiotherapy responsiveness, and enhancing its radiosensitivity through targeted therapy are expected to provide an important experimental basis for improving the effect of tumor radiotherapy.

In this study, we aimed to investigate the new features of CYLD, clarify the effect of CYLD-HDAC axis in NPC radiotherapy. And exploring whether class I HDAC inhibitors can enhance tumor radiotherapy.

## Results

### CYLD interacts and inhibits the activity of HDAC1/2

**Fig. 1:**
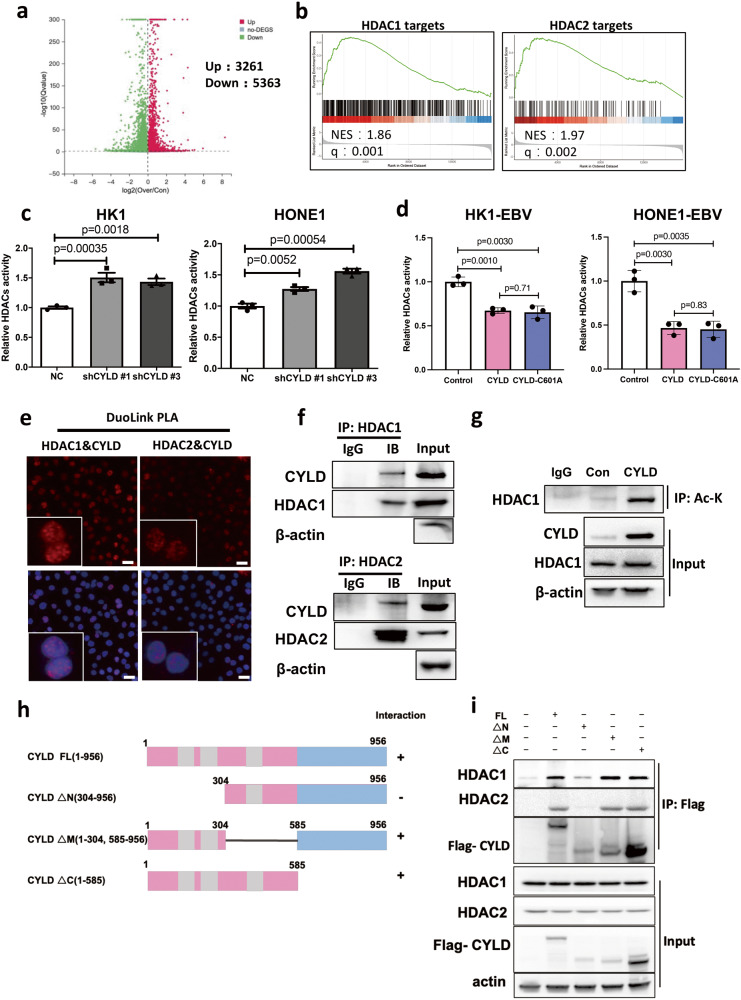
CYLD interacts and inhibits the activity of class I HDACs. **a** A volcano of differentially expressed genes identified by mRNA-seq in control and CYLD overexpressing NPC cells. **b** GSEA analysis of CYLD relative pathways. **c** HDACs activity measurement in control and CYLD knockdown cells. **d** HDACs activity measurement in control, CYLD, and CYLD-C601A overexpressing cells. **e** Proximity ligation assay indicating the interaction of CYLD and HDAC1/2 in HK1 cells (red: PLA positive signal; blue: DAPI, scale bar = 10 μm). **f** HONE1 cells were disrupted. The cell lysates were subjected to immunoprecipitation (IP) with anti-HDAC1/2. **g** HONE1-EBV cells transfected with the indicated plasmids, acetyl-Lys (Ac-K) was immunoprecipitated and probed with HDAC1 antibodies. The whole cell extract was used as input. **h** Schematic representation of the Flag-tagged full length CYLD (FL) and its various deletion mutants, including N-terminal 1-303 deletion (△N), middle domain deletion (△M), and C-terminal deletion(△C) constructs. **i** 293 T cells transfected with the indicated constructs were disrupted. The cell lysates were subjected to immunoprecipitation with anti-Flag.

NPC is an important subtype of head and neck squamous cell carcinoma, which is strongly driven by Epstein–Barr virus [14, 15]. Previously, we have reported that downregulated CYLD by EBV contributing to viral replication and NPC cell proliferation [4]. CYLD has also been found to promote apoptosis in NPC [25]. To further investigate the effects of CYLD in NPC, we performed RNA-seq analysis for HONE-EBV cells transfected with CYLD or an empty vector. CYLD expression caused significant changes in transcriptional profiles in HONE1-EBV cells, in which 3261 genes were upregulated and 5363 genes downregulated. Gene Set Enrichment Analysis (GSEA) was conducted for the differentially expressed genes in the CYLD overexpressing group and the control group (Fig. 1a). We focused on the top ten significantly altered pathways, which contain HDAC1 and HDAC2 related pathways (Fig. 1b). Additionally, we used a liquid chromatography coupled with tandem mass spectrometry (LC–MS/MS) approach to identify CYLD-binding proteins. Intriguingly, HDAC1 and HDAC2 were present in the purified CYLD complexes (Table S3, S4). These results indicated that CYLD may interact with and modulate the function of HDAC1 and HDAC2 complex. HDACs enzyme activity kit was used to further investigate whether the expression of CYLD mediates the deacetylase activity of HDACs. The enzymatic activity of HDACs was detected, and the results showed that the activity of HDACs was increased in CYLD knockdown cells (Fig. 1c). Also, the HDACs activity decreased significantly with the overexpression of CYLD (Fig. 1d). These results suggest that CYLD can inhibit the deacetylase activity of HDACs. Studies have found that CYLD regulated HDAC6 and HDAC7 in a non-enzymatic-dependent manner [26–28]. Thus, deubiquitinase function-deficient CYLD plasmid (C601A: cysteine 601 changed to alanine) was constructed and found that CYLD inhibits HDACs activity also in an enzymatic-independent manner. (Supplementary Fig. 1, Fig. 1d).

To further confirm whether CYLD binds to class I HDAC1 and HDAC2, we used PLA (proximity ligation assay). The results showed that CYLD interacts with HDAC1 and HDAC2 (Fig. 1e) and immunoprecipitation also showed that CYLD interacts with HDAC1/2 (Fig. 1f). HDAC1 and HDAC2 often coexist in multi-component protein complexes and are highly related enzymes [29]. Acetylation is the key post-translational modification of HDAC1, acetylation on HDAC1 represses its deacetylase activity and results in inactive deacetylase dimer. Thus, the acetylation level of HDAC1 was detected by immunoprecipitation (Fig. 1g). The results showed that overexpression of CYLD enhanced HDAC1 acetylation, which indicating CYLD inhibits HDAC1 activity. We next constructed a series of CYLD structural deletion mutants to investigate the main regions of the CYLD-HDAC interactions (Fig. 1h). After co-transfection with different plasmids, the Co-IP results showed that the N-terminal (1-303aa) of CYLD was mainly responsible for binding class I HDAC molecules (Fig. 1i).

### CYLD induces high oxidative stress via class I HDACs

**Fig. 2:**
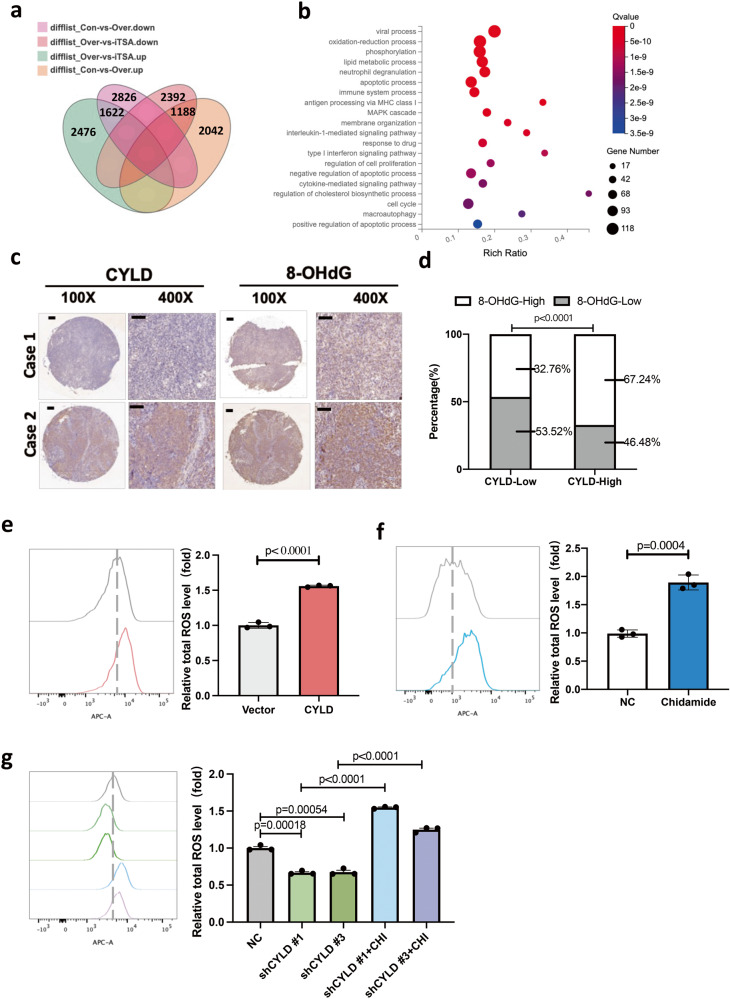
CYLD induces high oxidative stress by regulating HDACs. **a** Venn plot showing the overlap of differential genes from different treatment groups. **b** differential genes from Venn plots were enriched by GO. **c** Representative IHC photographs showing the expression of CYLD and 8-OHdG in consecutive sections of NPC microarrays. **d** 8-OHdG level is calculated based on CYLD expression in NPC microarrays. **e** ROS levels of control and CYLD overexpressing HONE1-EBV cells were detected by FCM by using CellROX Deep Red. **f** ROS levels of HONE1-EBV cells with or without Chidamide treatment (0.5 μM) were detected by FCM by using CellROX Deep Red. **g** ROS levels of CYLD knockdown HONE1 cells with or without Chidamide treatment (0.5 μM) were detected by FCM by using CellROX Deep Red.

RNA sequencing was used to further explore the functions of CYLD-HDAC axis. The analysis of the enriched differentially expressed genes revealed that HDACs are mainly involved in the oxidation-reduction process and in some metabolic processes (Fig. 2a, b). Next, an NPC tissue microarray was used to examine the level of 8-hydroxydeoxyguanosine (8-OHdG) by IHC. 8-OHdG is a reliable marker of oxidative stress-induced DNA damage. The results showed that CYLD is positively correlated with 8-OHdG levels (Fig. 2c, d). To determine whether CYLD-HDAC plays a causative role in inducing oxidative stress, the total intracellular ROS level was measured. The results showed that total ROS was notably increased by CYLD overexpression (Fig. 2e, Supplementary Fig. 2a). Class I HDACs inhibitor Chidamide significantly increased intracellular ROS levels (Fig. 2f, Supplementary Fig. 2b). In addition, Chidamide can effectively induce ROS accumulation in CYLD knockdown cells (Fig. 2g). This suggested that CYLD may induce toxic oxidative stress through inhibiting class I HDAC.

**Fig. 3:**
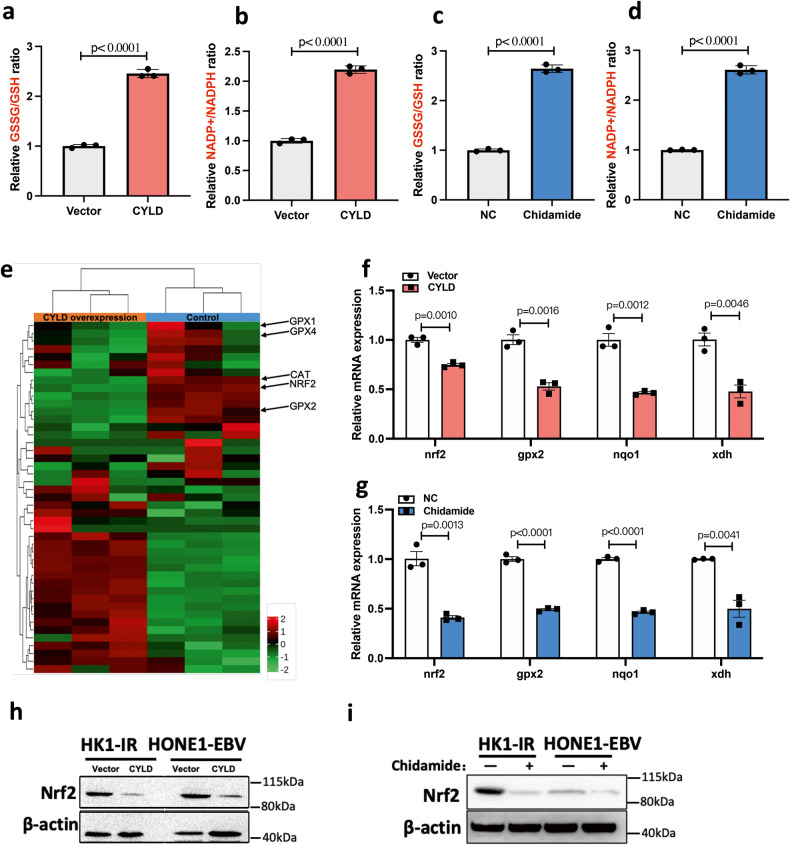
CYLD-HDAC axis regulates cell antioxidant activity. **a** Total glutathione (GSH) and oxidized glutathione (GSSG) of control and CYLD overexpressing HONE1-EBV cells were measured by using a GSH/GSSG assay kit. **b** Intracellular NADP + /NADPH levels of control and CYLD overexpressing HONE1-EBV cells were assayed by using an NADP + /NADPH assay kit. **c** Total glutathione (GSH) and oxidized glutathione (GSSG) of HONE1-EBV cells with or without Chidamide treatment (0.5 μM) for 24 h were measured by GSH/GSSG assay kit. **d** Intracellular NADP + /NADPH levels of HONE1-EBV cells with or without Chidamide treatment (0.5 μM) for 24 h were assayed by using an NADP + /NADPH assay kit. **e** A heat map showing the expression of the Redox-related genes across CYLD overexpression and control groups. **f** mRNA and **h** Protein level of indicate genes were detected after CYLD overexpressing in HONE1-EBV cells, β-actin was used as a control. **g** mRNA and **i** protein expression of indicated genes were detected with or without Chidamide treatment (0.5 μM) for 24 h in NPC cells. β-actin was used as a control.

ROS generation and elimination are the main oxidation-reduction processes in cells. Imbalance of ROS generation and elimination results in oxidative stress. We detected the ratios of the main redox pairs of GSSG/GSH and NADP^+^/NDAPH. The results showed that overexpression of CYLD notably increased the GSSG/GSH and NADP^+^/NDAPH ratios (Fig. 3a, b, Supplementary Fig. 3a, b). In addition, class I HDACs inhibitor Chidamide significantly increased the GSSG/GSH and NADP^+^/NDAPH ratios (Fig. 3c, d, Supplementary Fig. 3c, d). Further, we analyzed the CYLD overexpression RNA sequencing data, nuclear factor erythroid 2-related-factor 2 (Nrf2) and its downstream genes were found to be inhibited by CYLD expression (Fig. 3e). Nrf2 is the main transcriptional regulator of antioxidant responses. Nrf2 regulates the expression of key antioxidant enzymes, and the activation of Nrf2 plays a major role in ROS scavenging. Studies have found that class I HDACs enhance cellular antioxidant capacity through activating Nrf2 [30, 31]. We hypothesize that CYLD/HDAC axis regulates cellular antioxidant activity via Nrf2. Thus, we examined the mRNA and protein level of Nrf2 and its target antioxidant genes. These results showed that Nrf2 is downregulated after overexpression of CYLD and Chidamide treatment, Nrf2 target antioxidant genes gpx2, nqo1, and xdh were also downregulated (Fig. 3f–i, and Supplementary Fig. 3e, f). These results indicated that CYLD induces high oxidative stress by inhibiting cell antioxidant capacity.

### CYLD modulates DNA damage response through class I HDACs

**Fig. 4:**
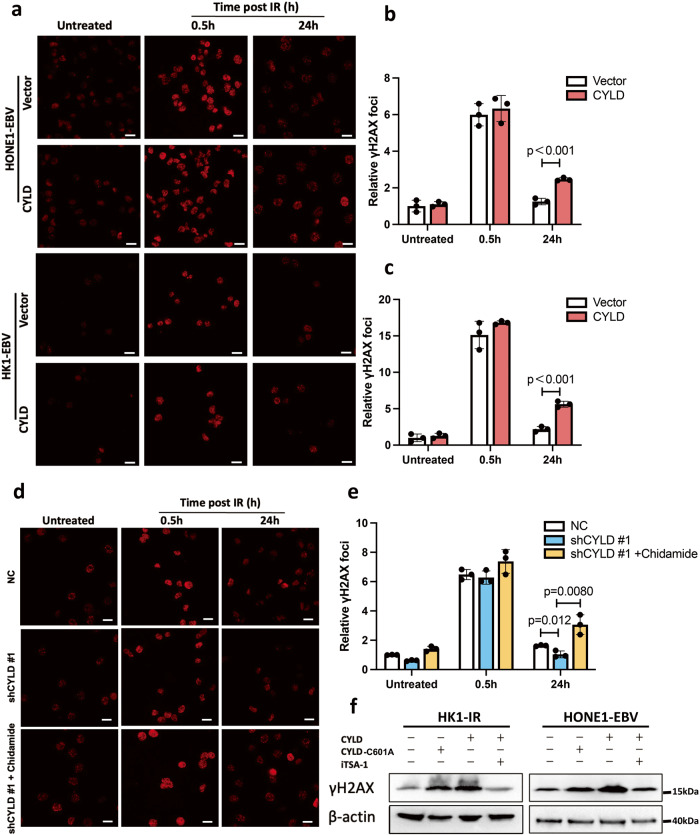
CYLD induces DNA damage through HDAC1/2. **a**–**c** Analysis of γH2AX foci at different times after 4 Gy radiation. Immunofluorescence staining for γ-H2AX followed by confocal microscopy was performed. Cells displaying 10 or more foci were counted as positive. Representative images of γH2AX foci and the percentage of HK1-EBV and HONE1-EBV cells displaying γH2AX foci are shown. **d**, **e** Immunofluorescence staining of γH2AX followed by confocal microscopy was performed. HONE1 cells by CYLD knockdown with or without Chidamide (0.5 μM) treatment for 24 h. **f** Detection of γH2AX protein level in NPC cells by CYLD overexpression with or without iTSA-1 (10 μM) for 24 h treatment.

HDAC1 and HDAC2 promote DNA double strand breaks (DSBs) repair by removing histone marker at DSBs, and HDAC1 deacetylation activity which is critical for its DSB repair function [32–34]. Since we have found that CYLD inhibits HDAC1 enzymatic activity. Therefore, we further to investigate whether CYLD-HDAC regulates DNA damage signaling. We tested the sensitive marker of DSBs, serine 139 of histone H2AX, which is quickly phosphorylated around the DSB site and is then referred to as γH2AX. As shown in Fig. 4a–c, CYLD overexpression groups have a higher level of γH2AX at 24 h after radiation. This result indicates that CYLD delayed DNA damage repair in NPC cells. Further, knockdown of CYLD increased DNA damage repair can be recovered by Chidamide (Fig. 4d, e). This indicates that CYLD inhibits DNA damage repair through class I HDACs. Furthermore, CYLD-induced DNA damage can be recovered by HDAC agonist iTSA-1 (Fig. 4f). These data indicates that CYLD negative regulates class I HDACs, leading to enhanced DNA damage response.

### CYLD-HDAC axis regulates radioresistance of NPC

Radiation is the main treatment strategy of NPC. Previously, we reported that low CYLD expression was significantly associated with recurrence and poor survival after radiotherapy in NPC patients [4]. Furthermore, patients who received radiotherapy were selected from the TCGA head and neck squamous cell carcinoma database. The evaluation specification effect of tumor treatment is evaluated by complete response (CR), partial response (PR), stable disease (SD), and progressive disease (PD). Based on standard, we defined CR & PR as radiotherapy sensitive and PD & SD as radiotherapy resistant for patients who have received radiotherapy. “High” and “low” groups were classified according to the median expression of CYLD. As shown in Supplementary Fig. 4a, the proportion of radiotherapy-sensitive (CR & PR) patients in the high CYLD-expressing group is about 10% higher than that in the low CYLD-expressing group (*p* < 0.05). These results indicate that the expression of CYLD may associated with radiotherapy sensitivity.

**Fig. 5:**
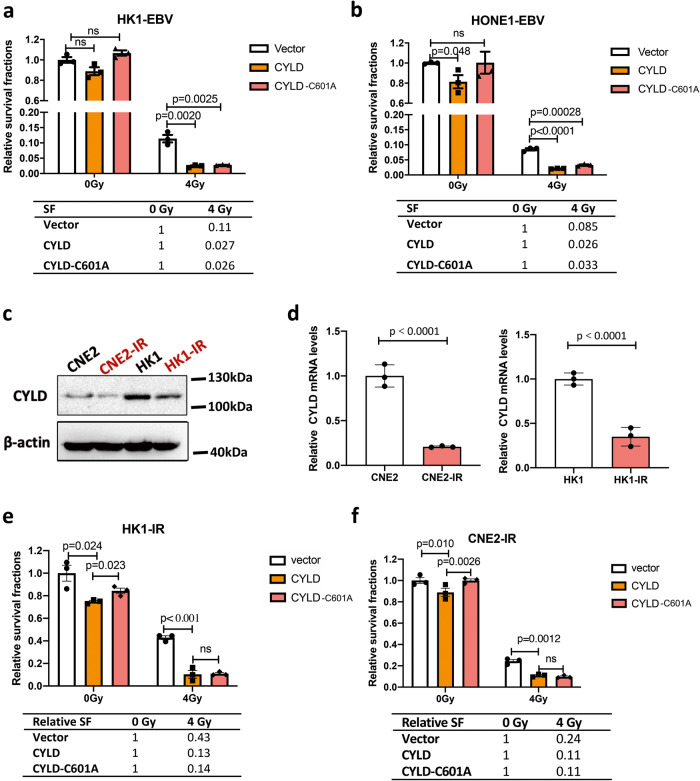
Downregulation of CYLD contributes to cancer radioresistance. **a** Immunoblot and **b** real-time PCR analysis of CYLD expression levels in radiation-resistant cells (CNE2-IR and HK1-IR) compared with radiation-responsive cells (CNE2 and HK1). β-Actin was used as a control. **c**–**f** Colony formation assay showing survival fractions of CYLD overexpression cells treated or not treated with 4 Gy irradiation, surviving fractions were calculated by comparing the colony number of each treatment group with untreated groups (0 Gy). (CYLD wt: full-length CYLD plasmid; CYLD-C601A: c601 mutant CYLD plasmid lacking enzyme function). The relative SF (survival fraction) is plotted below the results, Results are plotted as the mean surviving fraction ± SEM of 3 independent experiments.

Since EBV is a main cause of radioresistance in NPC [18, 35]. And CYLD was downregulated by EBV. We hypothesize that CYLD-HDAC may involve in the radio- resistant regulation of EBV-positive NPC cells. To test this hypothesis, CYLD was overexpressed in HK1-EBV and HONE1-EBV cells, which express very low levels of CYLD. Then we used a colony formation assay to evaluate radiation resistance of these cells. As predicted, after 4 Gy radiation, CYLD overexpression increased the sensitivity to radiotherapy (Fig. 5a, b). Further, the CYLD expression was examined in radiation-responsive (HK1, CNE2) and radiation-resistant (HK1-IR, CNE2-IR) NPC cells. Results indicated that the protein and RNA expression levels of CYLD are downregulated in radiation-resistant cells (Fig. 5c, d). Interestingly, the CYLD protein level was also reduced in radiation-resistant lung cancer cells (A549-IR, H358-IR) and radiation-resistant glioma cells (U251-IR) compared to radiation-responsive cancer cells (Supplementary Fig. 4b). These results indicate that CYLD downregulation may provide a common molecular basis for radiotherapy-resistant cells. As predicted, after 4 Gy radiation, CYLD overexpression increased the sensitivity to radiotherapy in radiation-resistant NPC cells (Fig. 5e, f). Notably, after 4 Gy radiation, both wild type and C601A plasmids expression of CYLD improve cellular sensitivity to radiotherapy. To further confirm the role of CYLD, stable CYLD knockdown cells were constructed by using a lentiviral-based CYLD small hairpin RNA. The survival fractions indicate that CYLD knockdown cells are more tolerant to radiation treatment than control cells (Supplementary Fig. 4c, d). These results confirmed that CYLD mediates radiosensitivity in an enzymatic-independent manner.

**Fig. 6:**
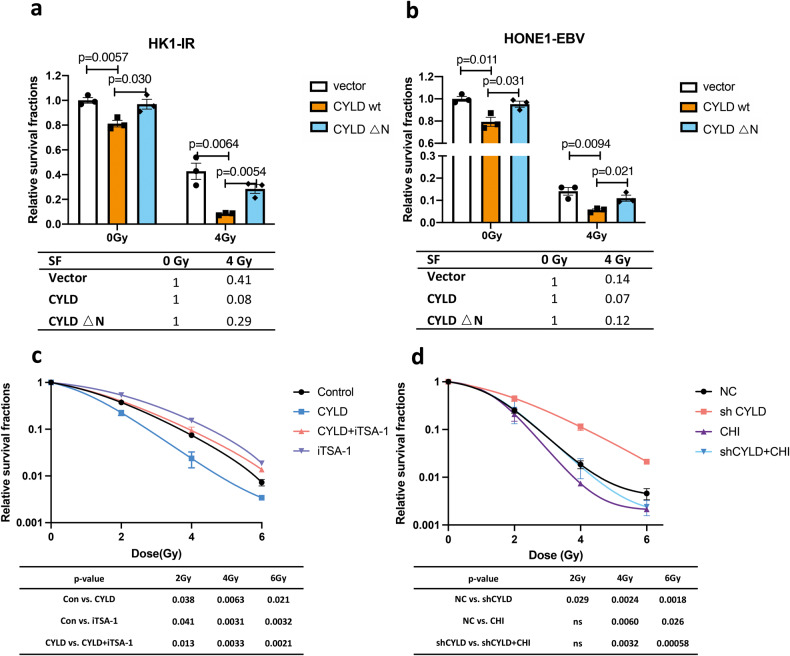
CYLD mediates radiation sensitivity through class I HDACs. **a**, **b** Colony formation assay showing survival fractions of CYLD overexpressing cells (CYLD wt: full-length CYLD plasmid; CYLD △N: N-terminal deletion CYLD plasmid) treated with irradiation or untreated; surviving fractions were calculated by comparing the colony number of each treatment group with untreated groups (0 Gy). **c** Colony formation assay showing survival fractions of CYLD overexpressing cells treated with iTSA (10 μM) or untreated at 24 h before irradiation; surviving fractions were calculated by comparing the colony number of each treatment group with untreated groups (0 Gy). **d** Colony formation assay showing survival fractions of CYLD knockdown cells, CHI (Chidamide: 0.5 μM) were treated 24 h before irradiation; surviving fractions were calculated by comparing the colony number of each treatment group with untreated groups (0 Gy). Results are plotted as the mean surviving fraction ± SEM of 3 independent experiments.

Radiotherapy triggers direct DNA damage to kill tumor cells and induces high oxidative stress conditions. Further, increased ROS kill cancer cells by induction of DNA damage and cell death pathways. Based on these, we further investigated whether CYLD promotes radiosensitivity by regulating class I HDACs. Firstly, wild-type and N-terminal defective CYLD plasmids were used. The results of a colony formation assay showed that N-terminal defective CYLD increased the survival fraction of radiation-resistant cells (Fig. 6a, b). These findings indicate that the N-terminal of CYLD mediates the radiosensitivity of cancer cells. Further, the cell survival fraction was calculated by counting the colony formation rate after a series of doses of radiation and the addition of the iTSA-1 to CYLD overexpressing cells. The results showed that the increased radiosensitivity caused by the overexpression of CYLD was reversed by iTSA-1 (Fig. 6c, Supplementary Fig. 5a). In addition, we added class I HDACs inhibitor Chidamide to the cells with stable knockdown of CYLD, the results indicated that CYLD knockdown-induced radiotherapy resistance was effective restored by Chidamide. The experimental results showed that the increased resistance to radiotherapy caused by the knockdown of CYLD was reversed (Fig. 6d, Supplementary Fig. 5b). These results indicated that CYLD induces NPC cells radiosensitivity through class I HDACs.

### The HDACs inhibitor Chidamide increases the radiosensitivity of NPC in vivo

**Fig. 7:**
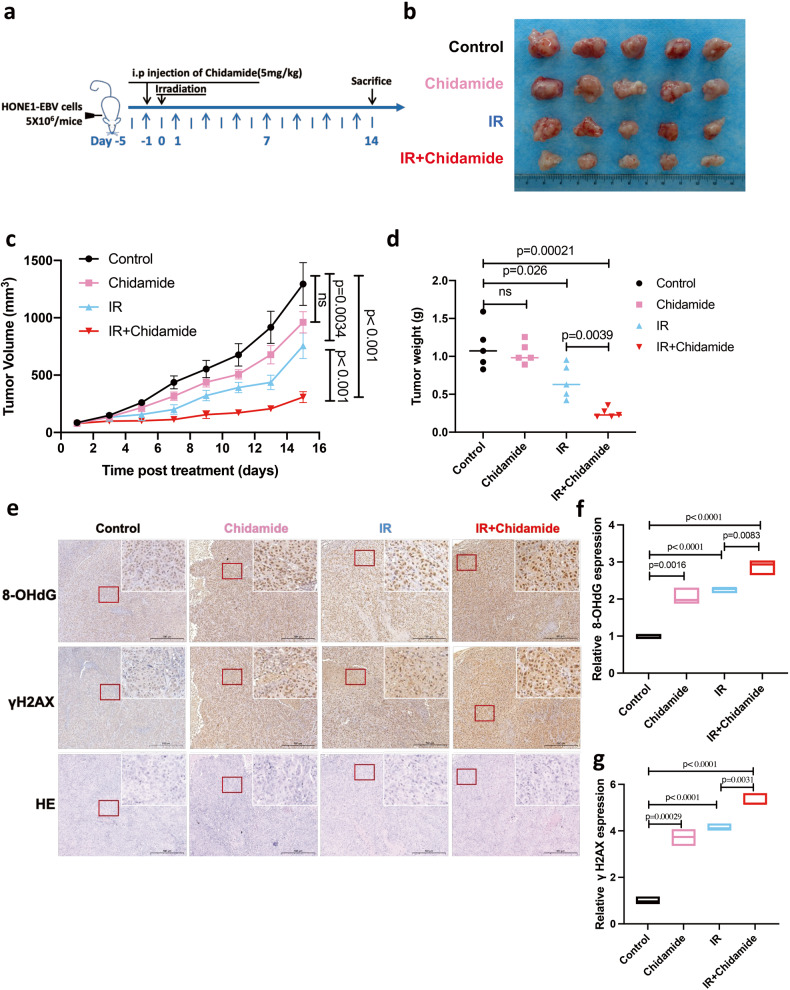
HDACi increases radiosensitivity in vivo. **a** The overall diagram of the study design. The NPC xenograft model was established using HONE1-EBV cells. **b** Representative images of xenografts from different treatment groups. Control: saline vehicle; Chidamide: Chidamide 5 mg/kg; IR irradiation with 4 Gy; IR + Chidamide: Chidamide-irradiation combination. **c** The tumor volume of xenograft from the indicated treatment group (*n* = 5). **d** Tumor weight of HONE1-EBV-derived xenografts from the indicated treatment group (*n* = 5). **e**–**g** Tumor sections were stained with hematoxylin and eosin (H&E) and subjected to immunohistochemistry detection for 8-OHdG and γH2AX (scale bar, 500 μm). Results are plotted as the mean surviving fraction ± SEM.

To further confirm the radio-sensitizing effect of HDACi in vivo, we treated athymic nude mice bearing HONE1-EBV xenografts with Chidamide, radiation (IR), or a combination of Chidamide and IR (Fig. 7a). Chidamide combined with radiation significantly reduced tumor growth (Fig. 7b–d). To determine whether the effect of combining radiation and Chidamide was synergistic (greater than the sum of the group effects), combination indexes (CI) were calculated. Nude mice treated with treated with the combination of radiation and Chidamide had CI value with 0.63, which indicating a synergistic effect (CI < 1). These results indicated that Chidamide sensitizes HONE1-EBV xenografts to radiation. IHC experiments showed that the 8-OHdG and γH2AX stained cells increased in the three groups receiving treatments, especially in combined therapy group, indicating that oxidative stress and DNA damage was promoted (Fig. 7e). Overall, in vivo data demonstrated that HDACs inhibition could increase the sensitivity of NPC to radiation.

### ZNF202 transcription inhibits the expression of CYLD

**Fig. 8:**
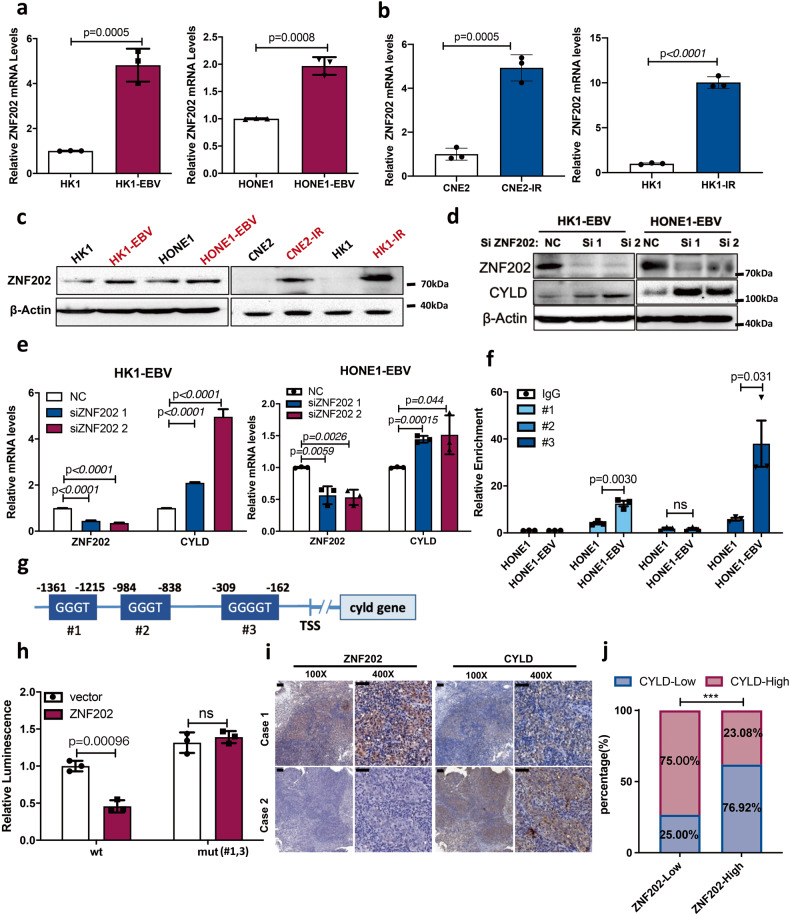
ZNF202 mediates transcriptional expression of CYLD. **a** Total RNA was isolated and subjected to real-time PCR analysis of ZNF202 in EBV-positive (HK1-EBV and HONE1-EBV) cells compared with EBV-negative (HK1 and HONE1) cells. **b** Total RNA was isolated and subjected to real-time PCR analysis of ZNF202 in radiation-resistant cells (CNE2-IR and HK1-IR) compared with radiation-responsive cells (CNE2 and HK1) cells. **c** Immunoblot analysis of ZNF202 in EBV-positive (HK1-EBV and HONE1-EBV) cells compared with EBV negative (HK1 and HONE1) cells and ZNF202 protein expression levels in radiation-resistant cells (CNE2-IR and HK1-IR) compared with radiation-responsive cells (CNE2 and HK1) cells. β-Actin was used as a control. HK1-EBV cells transfected with ZNF202 siRNAs: **d** Cell lysates were then extracted and subjected to Western blotting. β-Actin was used as a control. **e** total RNA from cells was isolated and subjected to real-time PCR. **f** The level of ZNF202 binding to the *CYLD* promoter in HONE1 and HONE1-EBV cells was analyzed by using ChIP followed by RT-PCR of 3 specific regions (*n* = 3). **g** Schematic illustration of the *CYLD* promoter and 3 potential binding sites of ZNF202. **h** 293 T cells were or were not co-transfected with ZNF202 and the luciferase reporter driven by the wild-type *CYLD* promoter or mutant *CYLD* promoter, together with a PLR-TK construct. Results are plotted as the mean surviving fraction ± SEM of 3 independent experiments. **i** Representative IHC staining of ZNF202 and CYLD expression from pathological sections of nasopharyngeal squamous cell carcinoma patients. **j** The CYLD protein expression level was calculated according to ZNF202 expression of nasopharyngeal squamous cell carcinoma patients. High- and low-expressing groups were classified according to median score.

To identify the upstream regulation mechanism of CYLD, we used the Genomatix (www.genomatix.de), JASPAR (jaspar.genereg.net), and University of California, Santa Cruz (UCSC; genome.ucsc.edu) databases to predict potential upstream transcription factors. In the analysis of the *CYLD* promoter region, we found that ZNF202 is a possible transcriptional repressor of CYLD, and 3 potential binding sites were identified (Fig. 8g). We also found that the mRNA and protein levels of ZNF202 were markedly increased in EBV-positive HK1-EBV and HONE1-EBV cell lines, as well as in the radiation-resistant (HK1-IR, CNE2-IR) NPC cells compared with HK1 and HONE1 cells (Fig. 8a–c). Higher protein levels of ZNF202 were also detected in NPC tissues compared to nasopharyngitis tissues (Supplementary Fig. 6a, b). The patients with high expression of ZNF202 (*n* = 71; median survival time: 60 months) had shorter overall survival (*n* = 58; median survival time: 96 months) and recurrence-free survival compared to patients expressing lower levels of ZNF202 (Supplementary Fig. 6c, d). To further investigate the association between ZNF202 and CYLD, small interfering RNA (siRNA) was used to knockdown ZNF202 in HK1-EBV and HONE1-EBV cells. Results showed that CYLD mRNA and protein levels were increased by knocking down ZNF202 (Fig. 8d, e). ChIP assays demonstrated that compared to HONE1 cells, ZNF202 binding to the CYLD promoter section 1 and 3 was increased in HONE1-EBV cells (Fig. 8f, g). We then generated luciferase reporter constructs driven by the wild-type *CYLD* promoter or promoters with deletion of these sites. Results showed that deletion of these sites markedly increased the *CYLD* promoter activity (Fig. 8h). These results suggested that ZNF202 inhibits the transcription of CYLD mainly by binding to these two sites. Next the relationship of ZNF202 and CYLD was analyzed by IHC in NPC tissues and a tumor tissue microarray. A significant negative correlation was observed in these NPC tissues (NPC patients: *r* = −0.43, NPC microarray: *r* = −0.42, Fig. 8i, j, and Supplementary Fig. 6e, f).

Furthermore, to determine whether the combination of ZNF202 and CYLD could be used as a prognostic factor, patients were classified into 4 groups based on median score of ZNF202 and CYLD as follows: Group1 (29/129) – low ZNF202 and CYLD expression; Group 2 (29/129) – low ZNF202 but high CYLD expression; Group 3 (45/129) – high ZNF202 but low CYLD expression; and Group 4 (26/129) – high ZNF202 and CYLD expression (Supplementary Fig. 6g). The four groups were further classified into three risk classes. Group1 and 2 are low risk, Group 3 is high risk, and Group 4 is intermediate risk (Supplementary Fig. 6h). Differences in overall survival and recurrence-free survival were significant among the four groups, and Group 3 had the worst prognosis (Supplementary Fig. 6i, j).

## Discussion

CYLD is an important suppressor in tumorigenesis and development. Previously, we have identified that CYLD inhibited cell cycle G1 to S phase transition by deubiquitinating p18, then negatively regulating cancer-cell proliferation [4]. In this study, we found that CYLD interacts with class I HDAC1 and HDAC2, and the N-terminus (1-303) of CYLD was found to be mainly responsible for binding to these HDACs. HDAC1 and HDAC2 share quite high homology [32]. As shown by MS results, the peptides of two HDACs bound on CYLD also have many identical or highly similar sequences. These findings indicate that CYLD interacts with either HDAC1 or HDAC2 because of their similar sequences. Enzyme activity assays showed that CYLD inhibited the activity of HDACs. Studies have identified that CYLD is able to inhibit the function of class II histone deacetylases HDAC6 and HDAC7 by interacting with their catalytic domain [26–28]. Our MS results also support this. Further, we constructed enzymatic-defective CYLD plasmid, and found that CYLD regulates class I HDACs also in an enzymatic-independent manner, which is consistent with the reported studies. Acetylation modification plays a decisive role in the activity of HDAC1 [36, 37]. Acetylation on HDAC1 represses its deacetylase activity and results in inactive deacetylase dimer [38]. These indicated that acetylation level is a key activity marker of HDAC1. HDAC activity assay showed that CYLD inhibits pan-HDACs activity. Further, acetyl- immunoprecipitation assay showed that CYLD inhibit the deacetylation activity of HDAC1. Since CYLD doesn’t work as an acetyltransferase or deacetylase, and the interaction is mediated via its N-terminal. This reminds us that the interaction may expose the acetylation sites of HDACs or promote its binding to acetyltransferases. Our MS result identified that CYLD interacts with Sin3A-associated protein 130 (SAP130), which is a Histone deacetylase complex subunit. Studies have identified that histone acyltransferase p300 interacts with Sin3A16, and Sin3A can form complex with HDAC, which form a dynamic regulating complex [39, 40]. It has been reported that p300 is the histone acyltransferase of HDAC1 [37]. These remind us that the interaction between CYLD and SAP130 may promote p300 binding to and acetylating HDAC1.

Class I HDACs, especially HDAC1 and HDAC2 strongly enhances antioxidant capacity. Studies have identified that class I HDACs deacetylates YB-1, which increasing its binding to 50-UTRs of NFE2L2 encoding the antioxidant factor NRF2, thereby enhancing NFE2L2 translation and synthesis of NRF2 to inhibit cellular ROS [31]. Nrf2 is the most important defense transcriptional factor against oxidative stress by transcription of antioxidant genes. Studies have found that HDAC inhibitors kill aggressive malignancies and shrink tumors by converging on the TXNIP/thioredoxin antioxidant pathway. In addition, several class I HDACi inhibits Nrf2 expression and induces oxidative stress [30, 31]. These studies suggest that Nrf2 is the core molecule in HDAC-mediated antioxidant function. We identified that class I HDACi Chidamide inhibits Nrf2 and its target antioxidant genes expression. Further we found that CYLD induces cellular oxidative stress by destroying HDAC-mediated Nrf2 antioxidant process.

Therapeutic efficacy is limited by a wide variety of cytoprotective pathways, which can be activated by radiotherapy. The DNA DSB is a main lesion responsible for the aimed cancer-cell killing in radiotherapy. In addition, the production of ROS is also the main event in radiotherapy [41]. ROS accumulation is a powerful inducer to DNA damage [42, 43]. Suppression of ROS and upregulation of DNA damage responses are as major mechanisms of radioresistance to standard treatment across cancers. Study has reported that CYLD as a deubiquitinase facilitating p53-dependent DNA damage [44]. In this work, we verified enzyme defective CYLD can also induce DNA damage. Evaluated ROS induced by CYLD contributes to DNA damage. More importantly, CYLD inhibits radiation-induced DNA damage repair (DDR) to enhance DNA damage induced by radiation. Studies have showed that class I HDACs directly participate in the DNA repair process. HDAC1 and HDAC2 localize to DNA damage sites, and deacetylate H3K56 and H4K16, which are required for the DDR, particularly through non-homologous end-joining (NHEJ) [32, 45]. Knockdown of HDAC1-3 impaired DNA repair, resulting in increased and persistent γH2AX, and hypersensitivity to ionizing radiation [34, 46]. Studies have confirmed that HDAC1 interacts with and deacetylates 8-oxoGDNA glycosylase 1 (OGG1), the key DNA oxidative damage repair enzyme, enhancing its cleavage activity [47, 48]. OGG1 knockdown and treatment of cells with OGG1 inhibitors sensitize cancer cells to radiation [49]. We found that class I inhibitor Chidamide enhance radiation-induced DNA damage response by suppressing DNA repair. CYLD-induced DNA damage can be recovered by iTSA-1. Thus, it is confirmed that CYLD can inhibits DNA damage repair via inhibiting class I HDACs. All of these indicates that CYLD regulates DNA damage response by inducing DNA damage and inhibiting DNA repair.

NPC is an infection-related cancer strongly driven by EBV [15]. EBV activate oncogenic signaling axes causing multiple malignant phenotypes and therapeutic resistance. Our group reported that EBV inhibits DNA-dependent protein kinase (DNA-PK) activity, and thus protects against radiation through DNA damage and oxidative stress [18, 50]. Here, we found that EBV enhances cell antioxidant capacity and DDR to protect cells from radiotherapy by inhibiting CYLD expression. To clarify the possible implications of downregulation of CYLD, we predicted and confirmed that ZNF202 is a transcriptional suppressor of CYLD. High ZNF202 levels were significantly related to poor survival and increased risk of recurrence. Further, we identified EBV downregulated CYLD via transcriptional suppressor ZNF202. These suggested that the viral abduction host mechanism to promote tumorigenesis and treatment resistance. Notably, CYLD is also downregulated in radiation-resistant NPC, glioma, and lung cancer cells. Therefore, downregulation of CYLD expression seems to be one of the important characteristics of radiotherapy resistance.

HDAC inhibitors (HDACi) can counteract the abnormal level of acetylation of tumor cell proteins. In recent years, specific or non-specific HDACi has been used for tumor treatment research at the preclinical levels either alone or in combination with chemoradiotherapy; and several HDACi have now entered the clinical research stage [12, 51–53]. HDACi panobinostat promotes MRE11 degradation, which in turn promotes radiosensitivity of bladder cancer cells by enhancing DNA damage [54]. HDACi combined with IKK inhibitors can enhance the therapeutic effect against solid tumors including ovarian cancer [55]. Furthermore, the combination of HDACi and immunotherapy significantly promote antitumor immunity [56]. We have identified the function of the CYLD-HDAC axis in radiotherapy and HDACi can increase the sensitivity of cancer cells and tumors to radiation therapy both in vitro and in vivo. Patients might benefit from the combined therapy of HDACi and radiation, thereby providing novel perspectives for the precise treatment of cancer.

**Fig. 9:**
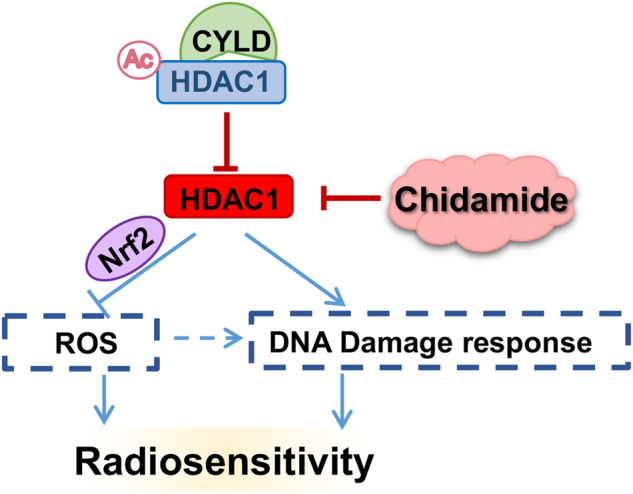
CYLD induces high oxidative stress and DNA damage through class I HDACs to promote radiosensitivity in nasopharyngeal carcinoma. We discovered a mechanism which CYLD binds to and inhibits class I HDACs enzyme functions by inducing HDAC1 acetylation. While class I HDACs mediate redox abnormalities and DNA damage repair, which leading to radiotherapy resistance in CYLD low-expressing tumors. Blocking HDACs by class I HDACs inhibitor Chidamide could effectively decrease radioresistance in vitro and in vivo. HDACi Chidamide could be a promising therapeutic strategy in CYLD low-expressing tumors to increase tumor radiotherapy sensitivity. (Ac acetylation, HDAC1(blue background: inactive; red background: active)).

This work focus on the modulation of class I HDACs and elucidate the novel function of CYLD in the non-enzymatic-dependent mediation of HDACs activity. Then, we identified that CYLD inhibits class I HDACs activity, which increasing oxidative stress and DNA damage, thereby promoting tumor radiosensitivity. We report the mechanisms of HDACs in the mediation of tumor radiotherapy resistance and explore the possibility of targeting class I HDACs to increase NPC radiotherapy sensitivity. Finally, we provide a novel molecular target and possible therapeutic strategy for tumor radiotherapy (Fig. 9).

## Materials and methods

### Cell culture

The human NPC cell lines, HK1, HK1-EBV, HONE1, and HONE1-EBV, were generously provided by Professor Sai Wah Tsao from the University of Hong Kong. The radiation-resistant NPC cells, CNE2-IR and HK1-IR were established as described previously [57, 58]. Cells were irradiated at a dose rate of 2 Gy/min using the X-RAD255 (Precision X-ray, North Branford, CT). Cells were cultured in 37 °C incubators with 5% CO_2_. Radiation-resistant GBM cells line U251-IR was generated with fractionated doses (2 Gy × 20) of radiation [59]. Radiation-resistant lung cancer cells A549-IR and H358-IR were generously provided by Professor Xingming Deng from Emory University School of Medicine. U251 cells were cultured in DMEM medium (Cat: 11995065, Gibco, Grand Island, USA) supplemented with 10% fetal bovine serum (Cat: BS-1105, Inner Mongolia Opcel Biotechnology Co., Ltd). The rest of the cells were cultured in RPMI-1640 medium (Cat: 11875500, Gibco, Grand Island, USA) supplemented with 10% FBS.

### Reagents and antibodies

LipoMax plasmid transfection reagent (Cat. 18101223) was purchased from SUDGEN (Bellevue, WA, USA). Dynabeads (Cat. 10002D) were obtained from ThermoFisher (Waltham, MA, USA) and RIPA buffer (Cat. P0013) was from Beyotime (Shanghai, China). ITSA-1 was purchased from MedChemExpress (MCE) (Cat. HY-100508). Chidamide was purchased from Selleck (Cat. S8567). Anti-mouse IgG-HRP (Cat. SC-2005) and anti-rabbit secondary antibody (Cat. SC-2004) were purchased from Santa Cruz BioTechnology (California, USA). Anti-CYLD was purchased from Cell Signaling Technology (Cat: 8462 S, CST, Boston, USA) and Abcam (Cat: ab137524, Cambridge, MA, USA). Anti-HDAC1 was purchased from CST (Cat:5356, and 34589). Anti-HDAC2 was purchased from CST (Cat:57156). Anti-Flag was purchased from Sigma–Aldrich (Cat: F3165) and Proteintech (Cat: 80010-1-RR, Wuhan, China). Anti-Myc was purchased from CST (Cat: 3946). Anti-pan acetylation antibody was purchased from Proteintech (Cat: 66289-1-Ig). Anti-8-HIydroxy-2′-deoxyguanosine (ab48508) and anti-Nrf2 (Cat: ab137550) were obtained from Abcam. Anti Phospho-Histone H2A.X (Ser139) (Cat: 20E3) was purchased from CST. (Cat: 9718). All antibodies were used according to the dilution ratio in the instructions.

### Plasmids and lentivirus transduction

The full-length and deletion constructs of CYLD were synthesized by GeneChem (Shanghai, China). shRNA targeting CYLD lentiviruses was purchased from GenePharma (Shanghai, China). The shRNA target sequences used included: CYLD sh#1, GCGTGTGTTGAAAGTACAATT; and CYLD sh#3, GCTGTAACTCTTTAGCATTTG. The catalytic inactive CYLD-C601A plasmid was changed cysteine 601 changed to alanine. The siRNA targeting ZNF202 was purchased from GenePharma (Shanghai, China).

### Clinical specimens

Nasopharyngitis and NPC tissues were collected from the Department of Pathology at Xiangya Hospital, Central South University, Changsha, China. The study was approved by the Medical Ethics Committee of Xiangya Hospital, Central South University (No. 201803134). The NPC tissue array (*n* = 129) was purchased from Outdo Biotech (HNasN129Su01, Shanghai, China). All clinical specimens were collected from biopsy before radiotherapy. The radiation of patients received was X-Ray. Immunohistochemical analysis was conducted as described previously [4].

### RNA sequencing (RNA-seq) and bioinformatic analysis

Total RNA was isolated from cells transfected with CYLD or treated with HDAC agonist iTSA, and then sent to Beijing Genomics institution for sequencing analysis. Gene abundance was calculated per kilobase of exon per million reads mapped (FPKM). The cDNA library was prepared for sequencing on a DNBSEQ sequencing platform. And then the data were analyzed by the online tool, Dr. Tom (https://biosys.bgi.com/).

### LC–MS/MS

Clarified lysates from 10^7^ cells was incubated overnight at 4 °C with 2 μg of specific primary antibodies or an isotype-matched negative control IgG. Subsequently, the samples were incubated for 1 h with 200 μl of magnetic beads conjugated with protein G (Invitrogen catalog no. 10004D) and then washed three times with IP wash buffer. They were then subjected to liquid chromatography coupled with tandem mass spectrometry (LC–MS/MS) analysis (Bioprofile, Shanghai, China).

### HDACs activity measurement

Enzymatic activity of HDACs was assessed by HDAC Activity Fluorometric Assay Kit (Cat: K330-100, Biovision) according to the manufacturer’s instructions. Briefly, 10–50 μg cell lysate was diluted to 85 μl (final volume) of ddH_2_O in each well. 10 μl of the 10X HDAC Assay Buffer was added to each well. Then 5 μl of the HDAC Fluorometric Substrate was added to each well and mixed thoroughly. Plates were incubated at 37 °C for 30 min. The reaction was stopped by adding 10 μl of Lysine Developer and then mixed. The plate was incubated at 37 °C for 30 min and samples read in a fluorescence plate reader (Ex/Em = 350–380/440–460 nm). The fluorescence value was detected by multi-functional enzyme labeling instrument (PerkinElmer VICTOR™ X3, USA).

### Proximity ligation assay (PLA)

Interacting proteins were detected by the DuoLink® In Situ Red Starter Kit Mouse/Rabbit (DUO92101, Sigma–Aldrich, Darmstadt, Germany). Cells were seeded in 8-well chamber slides (Millicell EZ SLIDE, Millipore, Darmstadt, Germany) and cultured overnight. Experimental steps were performed according to the manufacturer’s protocol and conducted as described previously [60]. Fluorescence images were acquired using a Leica TCS SP8 confocal microscope.

### Western blotting

Whole-cell lyses was extracted in RIPA lysis buffer with protease inhibitor cocktail (HY-K0010, MCE) on ice for 1 h, then centrifuged for 15 min at 13,200 × *g* at 4 °C. After determination of protein concentration by BCA method. 50 μg of protein were separated by 10% SDS-PAGE and transferred onto PVDF membranes (Millipore). After blocking, the blots were incubated with the specific primary antibody at 4 °C overnight and with HRP-conjugated anti-mouse or anti-rabbit secondary antibody for 2 h. Visualization was performed using the ChemiDoc XRS system and Image Lab software (Bio-Rad, CA, USA). Western blots were derived from the same experiment and processed in parallel. Uncropped scans of the Western blots are provided in Supplementary Materials.

### GSSG/GSH measurements

Reduced glutathione (GSH) and oxidized glutathione (GSSG) were measured using a GSH/ GSSG assay kit (Cat: G263, Dojindo, Japan) according to the manufacturer’s protocol. Cells were centrifuged for 10 min at 200 × *g* and the supernatant fraction was discarded. Then 80 μl 10 mM HCl were added, and the cells were cleaved by repeated freezing and thawing twice. 20 μl of 5% SSA was added, and sample centrifuged at 8000 × *g* for 10 min. The supernatant fraction was transferred to a new tube and diluted with ddH_2_O, and the concentration of SSA adjusted to 0.5%. Then 40 μl GSSG sample and GSH sample was added. 60 μl of Buffer Solution was added to each well and incubated at 37 °C for 1 h. Then 60 μl of Substrate Working Solution was added to each well. Subsequently, 60 μl Enzymes/Coenzymes Working Solution, the absorbance was measured at 405 nm or 415 nm after incubating at 37 °C for 10 min. The total protein concentrations of samples run in parallel were measured using a BCA protein assay kit (BBI, China). The total glutathione concentration was normalized to protein content.

### NADP^+^/NADPH measurements

Intracellular NADP^+^/NADPH levels were assayed using an NADP^+^/NADPH assay kit (Cat: N510, Dojindo, Japan) The total protein concentrations of samples run in parallel were measured using a BCA protein assay kit (BBI, China). The total NADPH concentration was normalized to protein content. First, collecting cells in 500 μl PBS, then 300 μl NADP/NADPH Extraction Buffer was added into each sample. After centrifugation for 5 min at 12,000 × *g*, transferring the supernatant to the MWCO 10 K ultrafiltration tube, centrifugation for 10 min at 12,000 × *g*. Then the total NADP^+^/NDAPH and NADPH level were measured according to the manufacturer’s instructions.

### Tumorigenicity assay

The tumorigenicity study was approved by the Medical Ethics Committee (for experimental animals) of Xiangya Hospital, Central South University (No. 201803135). Then 5 × 10^6^ cells per animal were injected subcutaneously into the flank regions of nude mice (BCLB/c-nu, female, 5 weeks old). The tumors were measured every other day and tumor volume was calculated using the following formula: V = (π × length × width^2^)/6. When tumors grew to an average volume of 150 mm^3^ prior to initiation of therapy. The mice were randomly assigned into 4 groups (*n* = 5) as follows: (1) Control: saline vehicle (100 μl); (2) Chidamide: Chidamide 5 mg/kg (100 μl); (3) IR: irradiation with 4 Gy; (4) IR + Chidamide: Chidamide (5 mg/kg, 100 μl) plus irradiation with 4 Gy. At the end of experiments, mice were euthanized by CO_2_ inhalation, and the weight of extracted xenograft tumors was obtained at the same time.

All animal experiments were approved by The Medical Ethics Committee of Xiangya Hospital and were performed at the animal experiment center of Xiangya Medical College, Central South University. Mice were housed under pathogen-free conditions with ad libitum food and water.

### Combination index (CI)

The combination index (CI) was calculated by using the Chou– Talalay equation. The general equation for the classic isobologram (CI = 1) was conducted as described previously [61]. CI < 1 indicates synergism, CI = 1 indicates an additive effect, and CI > 1 indicates antagonism.

### Chromatin immunoprecipitation (ChIP)

ChIP assays were performed using Chromatin Immunoprecipitation Kits (Catalog # 17-10085, Millipore). Cells were plated in a 100-mm dish and 24 h later, cells were fixed in 1% formaldehyde for 10 min and then quenched with glycine for 5 min. Cells were washed twice and scraped with 1 ml cold PBS and then centrifuged at 800 × *g* at 4 °C for 5 min to pellet and resuspended in 0.5 ml of cell lysis buffer. The supernatant fraction was removed being careful not to disturb the cell pellet and then cell pellets were resuspended in 0.5 ml of nuclear lysis buffer. Cell lysates were sonicated to shear DNA to 200–1000 bp and insoluble material was removed. Subsequent steps were performed according to the manufacturer’s protocol. Finally, DNA was purified with spin columns and eluted with 20 ml of ddH_2_O and subjected to qPCR analyses.

### Luciferase reporter assay

Luciferase activities were detected using Dual-Luciferase Reporter Kit (E1910, Promega) following the manufacturer’s recommendations. Cells were transfected as firefly luciferase reporter gene plasmid: sea kidney luciferase reporter gene plasmid = 50:1. After 48 h, cells were cleaved by Passive Lysis Buffer (PLB) 100 μl. 15–30 min, collecting the pyrolysis liquid. First measurement: add PLB 20 μl into the plate with light transmission at the bottom. Add Luciferase Assay Buffer II 100 μl to avoid light. The first fluorescence value is measured by chemiluminescence method. Second fluorescence value measurement: add 100 μl Stop&Glo reagent for the second fluorescence value measurement. Result analysis: Ratio = (first fluorescence value background fluorescence value)/ (second fluorescence value background fluorescence value).

### Statistical analysis

The experimental results were statistically evaluated using the Student’s *t*-test, the Pearson chi-square (*χ*^2^) test, One-way or two-way ANOVA, COX regression analysis, and Kaplan–Meier analysis. A value of *p* < 0.05 was considered statistically significant. All statistical analyses were performed using SPSS 26 or GraphPad Prism 9.0 software.

### Reporting summary

Further information on research design is available in the Nature Research Reporting Summary linked to this article.

### Supplementary information

Supplementary Materials

Reporting Summary

Original Data File

## Data Availability

The TCGA data referenced during the study are available in a public repository from TCGA website (http:// www.cbioportal.org/). Oncoprint (cBioPortal) for the HNSCC TCGA provisional cohort of tumors with complete data (sequencing, copy number alterations, and mRNA expression) showing tumors with percentages are relative to the complete number of tumors in the cohort (*n* = 279). The gene expression data of HNSCC patients is publicly available on the Cancer Genome Atlas (TCGA). Survival analyses and immunohistochemistry data are not publicly available to researchers. All other datasets generated during the study will be made available upon reasonable request to the corresponding author, Professor Ya Cao, email address: ycao98@vip.sina.com.
