# Supplementary material for: CYLD induces high oxidative stress and DNA damage through class I HDACs to promote radiosensitivity in nasopharyngeal carcinoma
Source: Cell Death Dis. 2024 Jan 29;15(1):95. doi: 10.1038/s41419-024-06419-w (PMC10824711; doi:10.1038/s41419-024-06419-w)
Supplement: Supplementary file 1 — Supplementary Materials [file 41419_2024_6419_MOESM1_ESM.docx]

**Supplemental Materials:**

**Methods:**

**RNA isolation and real-time PCR**

Total mRNA was isolated by using the NucleoZOL reagent (Cat: 740404, MACHEREY-NAGEL GmbH & Co. KG, Düren, Germany). The RevertAid First Strand cDNA Synthesis Kit (Cat: K1622, Invitrogen, Carlsbad, CA, USA) was used for reverse transcription. Real-time PCR analysis was performed in triplicate by using the SYBR^TM^ Green Master Mix (Cat: A25742, Invitrogen) and the ABI7500 Real-Time System (Applied Biosystems). Primers are listed in the table below.

| Gene | Forward Primer sequence | Reverse Primer sequence |
| --- | --- | --- |
| ZNF202 | CCTTCCTTCCTCAGTCACCA | CTAAAGAGACCGCGTTCCAG |
| CYLD | TGCCTTCCAACTCTCGTCTTG | AATCCGCTCTTCCCAGTAGG |
| Nrf2 | TACTCCCAGGTTGCCCACA | CATCTACAAACGGGAATGTCTGC |
| GPX2 | GGTAGATTTCAATACGTTCCGGG | TGACAGTTCTCCTGATGTCCAAA |
| NQO1 | CATTCTGAAAGGCTGGTTTGA | CTAGCTTTGATCTGGTTGTCAG |
| XDH | AGGTGGACCACTTCAGCAAT | GTTGGAGGGAAGGTTGGTTT |
| β-Actin | CCAAGGCCAACCGCGAGAAGATGAC | AGGGTACATGGTGGTGCCGCC AGAC |

**ROS level measurements**

Total intracellular ROS levels were assessed by using a CellROX Deep Red reagent for oxidative stress detection (Cat: C10422, Thermo Fisher Scientific, Waltham, MA, USA) according to the manufacturer’s instructions. Briefly, the cells were resuspended in PBS. The CellROX Deep Red reagent was added at a final concentration of 5 μM, and then incubated protection from light for 30 min at 37°C. After washing in PBS for 3 times, the cells were collected, and the CellROX Deep Red signal was detected using flow cytometry.

**Colony formation and radiosensitivity assays**

About 1500-3000 Cells were seeded in 6-well plates in triplicate. Then treating as indicated chemicals and/or exposed to different doses of irradiation. After incubating 7-14 days, cells were gently washed with PBS, fixed in methanol for 20 min and stained with crystal violet for 20 min at room temperature. Image J software was used to count colonies that contained more than 50 cells, and the survival fractions were calculated. Cells were irradiated at room temperature using X-ray Biological Irradiation Apparatus (X-RAD225, Precision X-ray) and 23EX Linear Accelerator (23EX, Varian Medical Systems). Cell viability fraction was calculated by the following formula: Clone formation rate = number of clones / number of cells seeded Survival fraction = clonal formation rate of irradiated cells / clonal formation rate of control cells.

**Immunoprecipitation**

Cells were disrupted in IP lysis buffer containing protease inhibitor cocktail and 1 mg protein aliquots were pre-cleared by incubating with 10 μl Dynabeads protein A (Invitrogen) for 1 hour at 4°C. The pre-cleared samples were incubated with antibody (2 μg/1mg sample) overnight at 4°C and then 20 μl Dynabeads protein A were added to samples and incubated for 2 hours at 4°C. The beads were washed 3 times with cold wash buffer, then boiled with 1X loading buffer and analyzed by Western blotting.

**Immunofluorescence analysis**

Cells were washed with cold PBS and fixed in 4% formaldehyde at room temperature for 20 min, and permeabilized for 15 min with 0.1% Triton X-100. Then the cells were blocked in 5% donkey serum for 1 hour at room temperature and incubated with a primary antibody in PBS with 1% BSA at 4°C overnight. The next day, cells were washed with cold PBS and incubated with a secondary antibody for 45 min at room temperature. Then cells were washed and stained with DAPI for 10 min and viewed by a confocal microscope (Leica TCS SP8, Germany) and Cell Imaging Microplate Detector (Biotek LionHeart LX).

**Tables**

**Table 1. The clinical characteristics of 20 NP and 29 NPC subjects**

**Table 2. The clinical characteristics of 129 NPC patients assessed by microarray**

**Table 3 Interacting sequences of CYLD and HDACs**

**
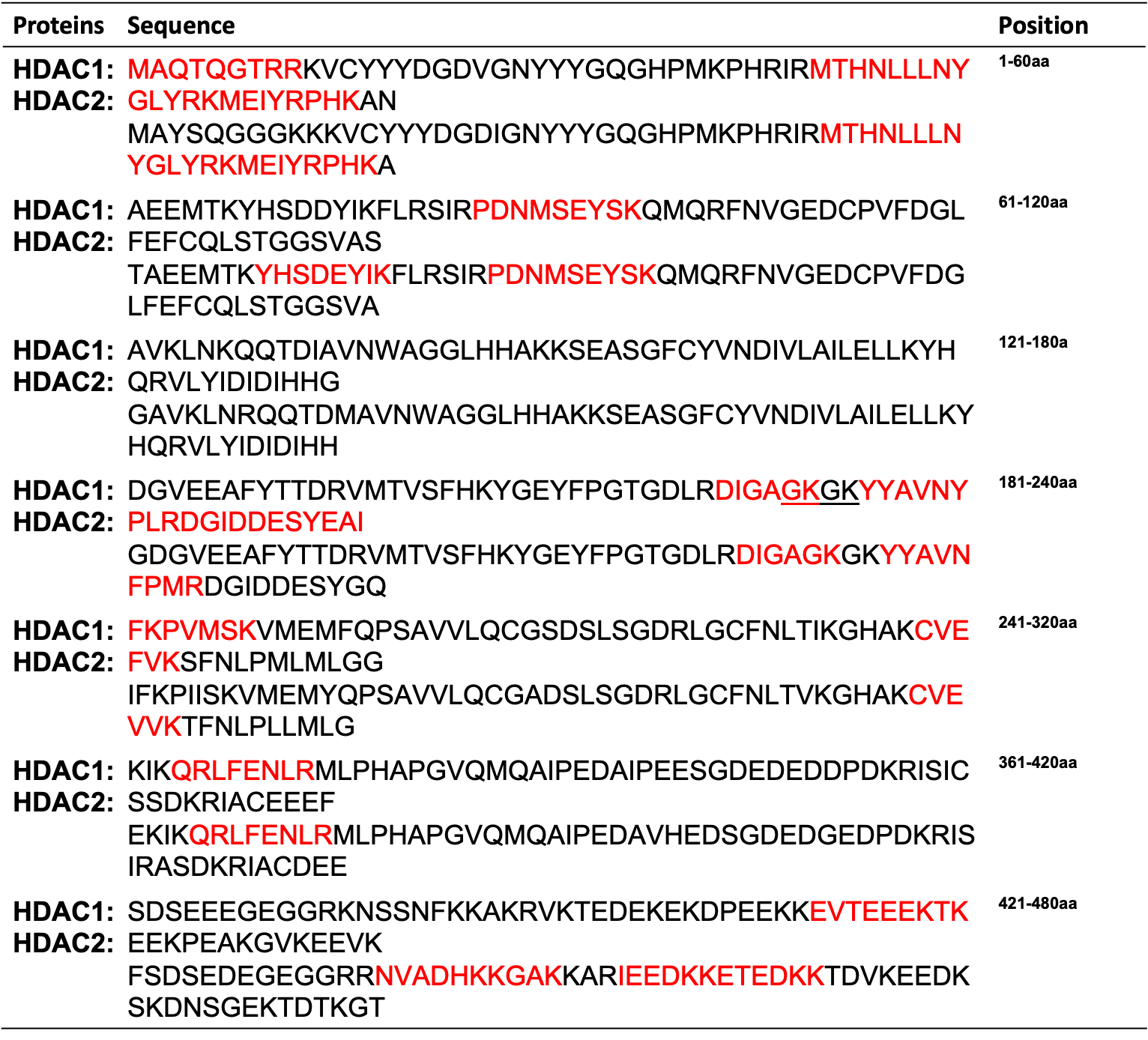
**

**Table 4 The top identified CYLD interacting proteins specifically in HONE1-EBV cells.**

| **Protein Name** | **Description** | **Protein Score** | **Unique peptide** |
| --- | --- | --- | --- |
| Kinesin-like protein KIF11 | KIF11 | 323.31 | 6 |
| Histone deacetylase 1 | HDAC1 | 323.31 | 11 |
| Transitional endoplasmic reticulum ATPase | VCP | 107.69 | 17 |
| Histone deacetylase 2 | HDAC2 | 91.54 | 16 |
| Thyroid hormone receptor-associated protein 3 | THRAP3 | 69.982 | 2 |
| Plakophilin-3 | PKP3 | 57.731 | 6 |
| BTB/POZ domain-containing protein KCTD5 | KCTD5 | 46.856 | 2 |
| Eukaryotic translation initiation factor 4B | EIF4B | 46.63 | 12 |
| THRAP3 protein (Fragment) | THRAP3 | 39.41 | 4 |
| ATPase family AAA domain-containing protein 3A | ATAD3A | 32.839 | 3 |
| …… |  |  |  |
| mSin3A-associated protein 130 | SAP130 | 13.98 | 1 |

**Supplemental figures:**

**
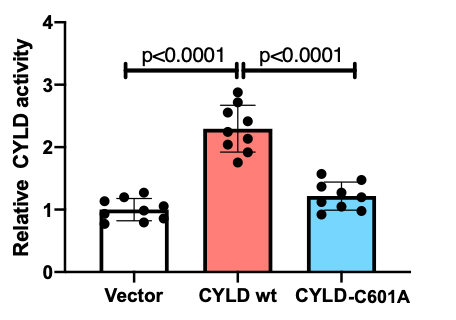
**

**Supplemental Figure 1** CYLD deubiquitinase activity measurement in control and CYLD overexpressing cells (CYLD wt: full length CYLD plasmid; CYLD-C601A: c601 mutant CYLD plasmid lacking enzyme function).


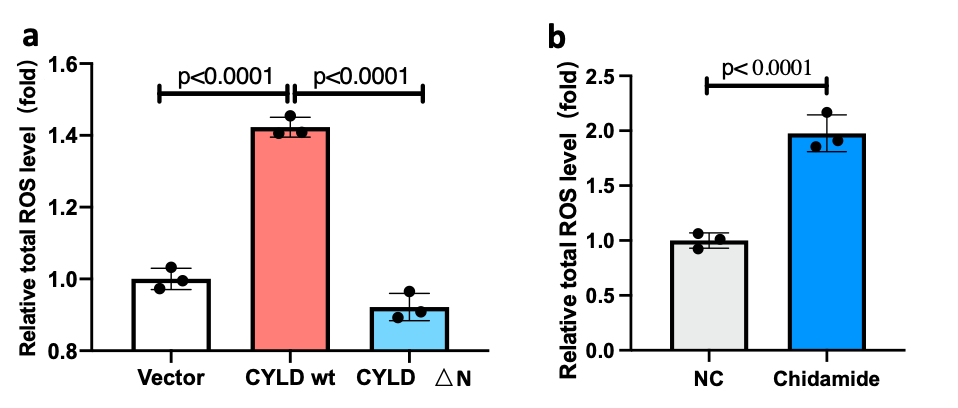


**Supplemental Figure 2.** **CYLD induces** **high oxidative stress by regulating HDACs.**

a) ROS levels of control and CYLD overexpressing (CYLD wt: full length CYLD plasmid; CYLD △N: N terminal deletion CYLD plasmid) in HK1-EBV cells were detected by FCM by using CellROX Deep Red. b) ROS levels of HK1-EBV cells with or without Chidamide treatment (0.5 μM) were detected by FCM by using CellROX Deep Red

**
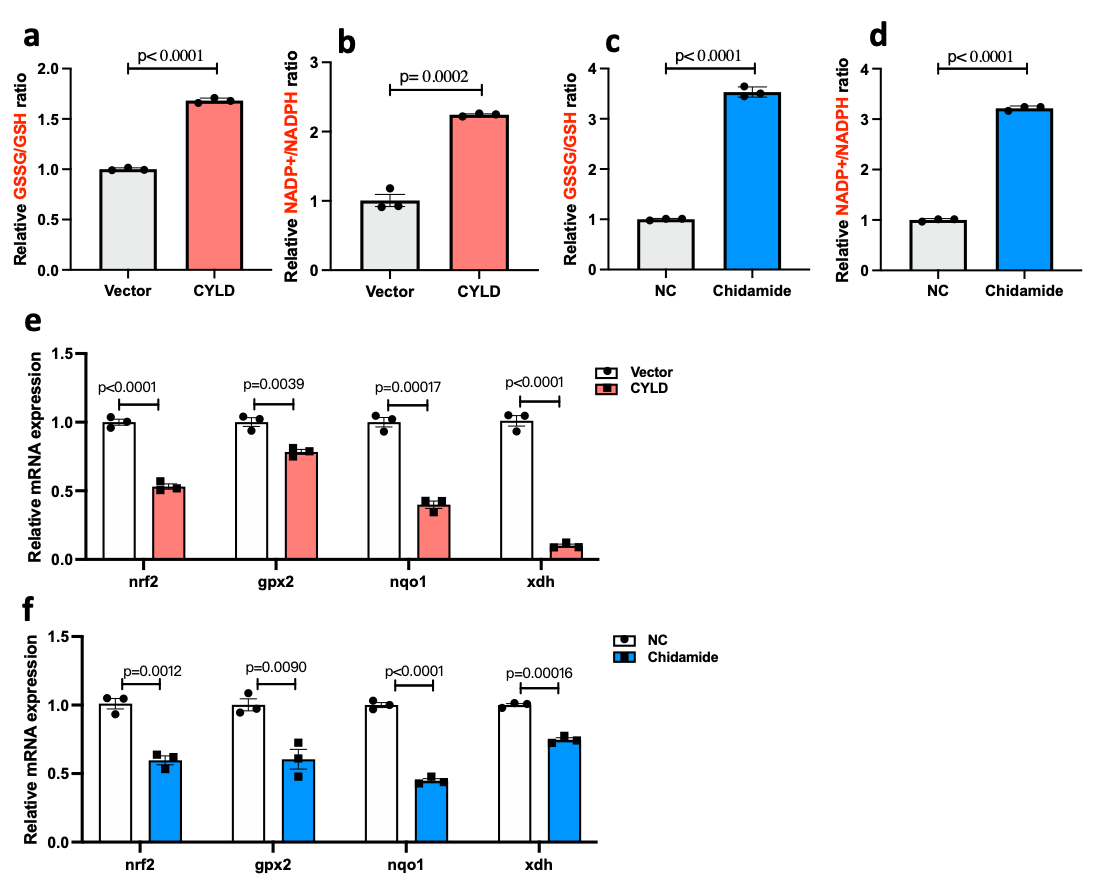
**

**Supplemental Figure 3. CYLD-HDAC axis regulates cell antioxidant activity.**

a) Total glutathione (GSH) and oxidized glutathione (GSSG) of control and CYLD overexpressing HK1-IR cells were measured by using a GSH/GSSG assay kit. b) Intracellular NADP+/NADPH levels of control and CYLD overexpressing HK1-IR cells were assayed by using an NADP+/NADPH assay kit. c) Total glutathione (GSH) and oxidized glutathione (GSSG) of HK1-IR cells with or without Chidamide treatment (0.5 μM) were measured by GSH/GSSG assay kit. d) Intracellular NADP+/NADPH levels of HK1-IR cells with or without Chidamide treatment (0.5 μM) were assayed by using an NADP+/NADPH assay kit. e) mRNA level of indicate genes were detected after CYLD overexpressing in HK1-IR cells, β-actin was used as a control. f) mRNA expression of indicated genes were detected with or without Chidamide treatment (0.5 μM) in HK1-IR cells. β-actin was used as a control.

**
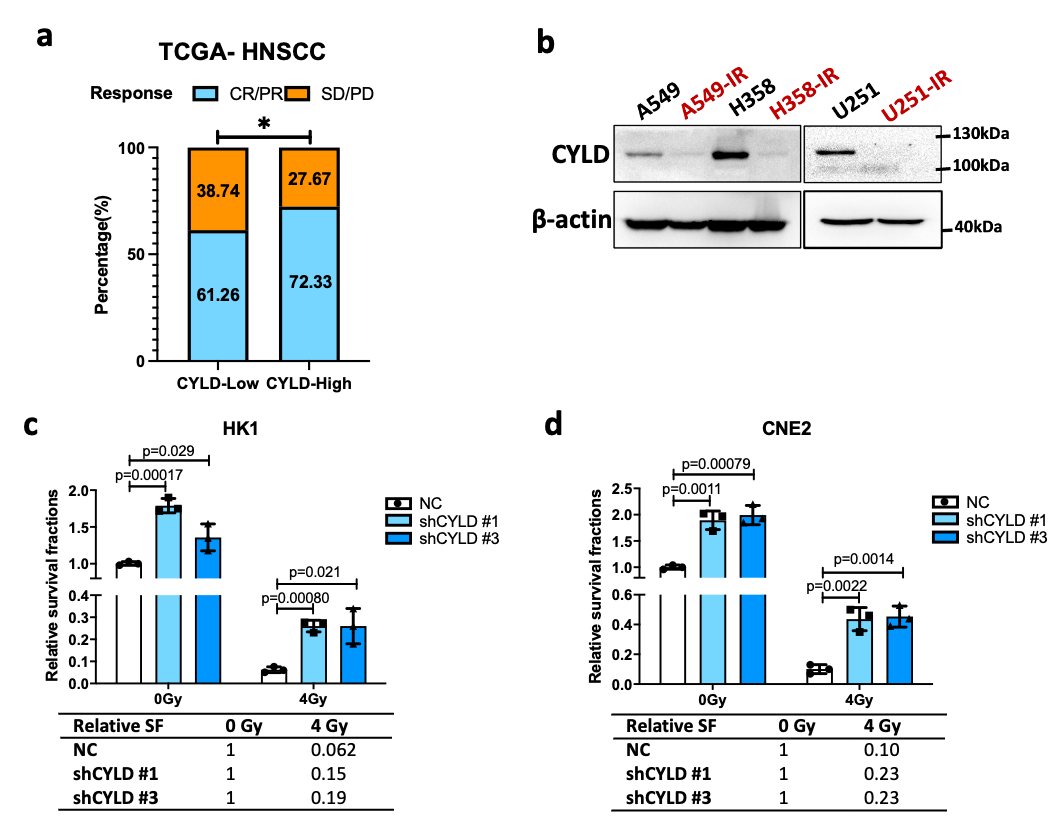
**

**Supplemental Figure 4. CYLD is downregulated in radiation-resistant cells.**

a) The treatment effect was evaluated by complete response (CR), partial response (PR), stable disease (SD), and progressive disease (PD). CR & PR as radiotherapy sensitivity and PD & SD as radiotherapy resistance for patients who have received radiotherapy. High and low groups were based on median score (**p* < 0.05). b) CYLD protein expression levels in radiation-resistant cells (A549-IR, H358-IR and U251-IR) compared with radiation-responsive cells (A549, H358 and U251) cells. β-Actin was used as a control. c, d) Colony formation assay showing survival fractions of CYLD knockdown cells treated or not treated with 4Gy irradiation; surviving fractions were calculated by comparing the colony number of each treatment group with untreated groups (0 Gy).

**
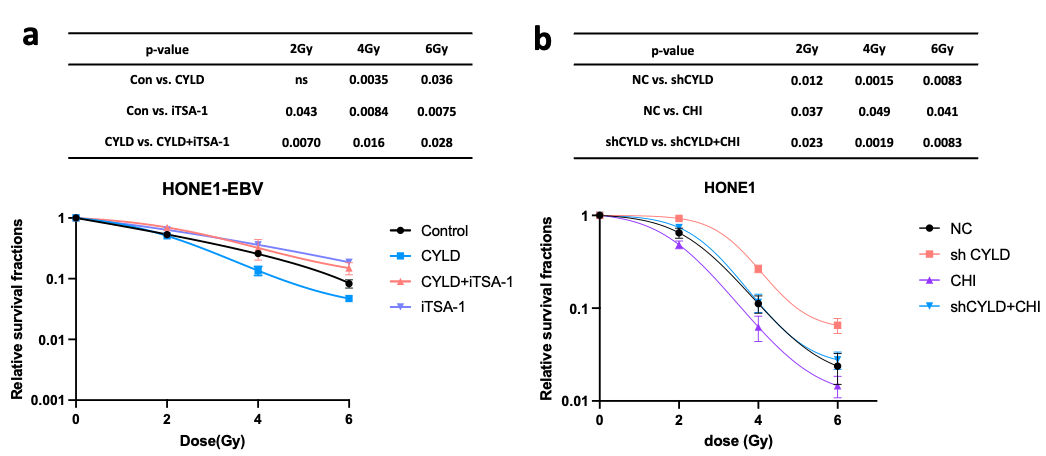
**

**Supplemental Figure 5. CYLD regulates radiation sensitivity through HDACs.**

a) Colony formation assay showing survival fractions of CYLD overexpressing cells treated or not treated with iTSA (10 μM) at 24 h before irradiation; surviving fractions were calculated by comparing the colony number of each treatment group with untreated groups (0 Gy). b) Colony formation assay showing survival fractions of CYLD knockdown cells. CHI (Chidamide: 0.5 μM) was added 24 h before irradiation; surviving fractions were calculated by comparing the colony number of each treatment group with untreated groups (0 Gy). Results are plotted as the mean surviving fraction ± SEM of 3 independent experiments.

**
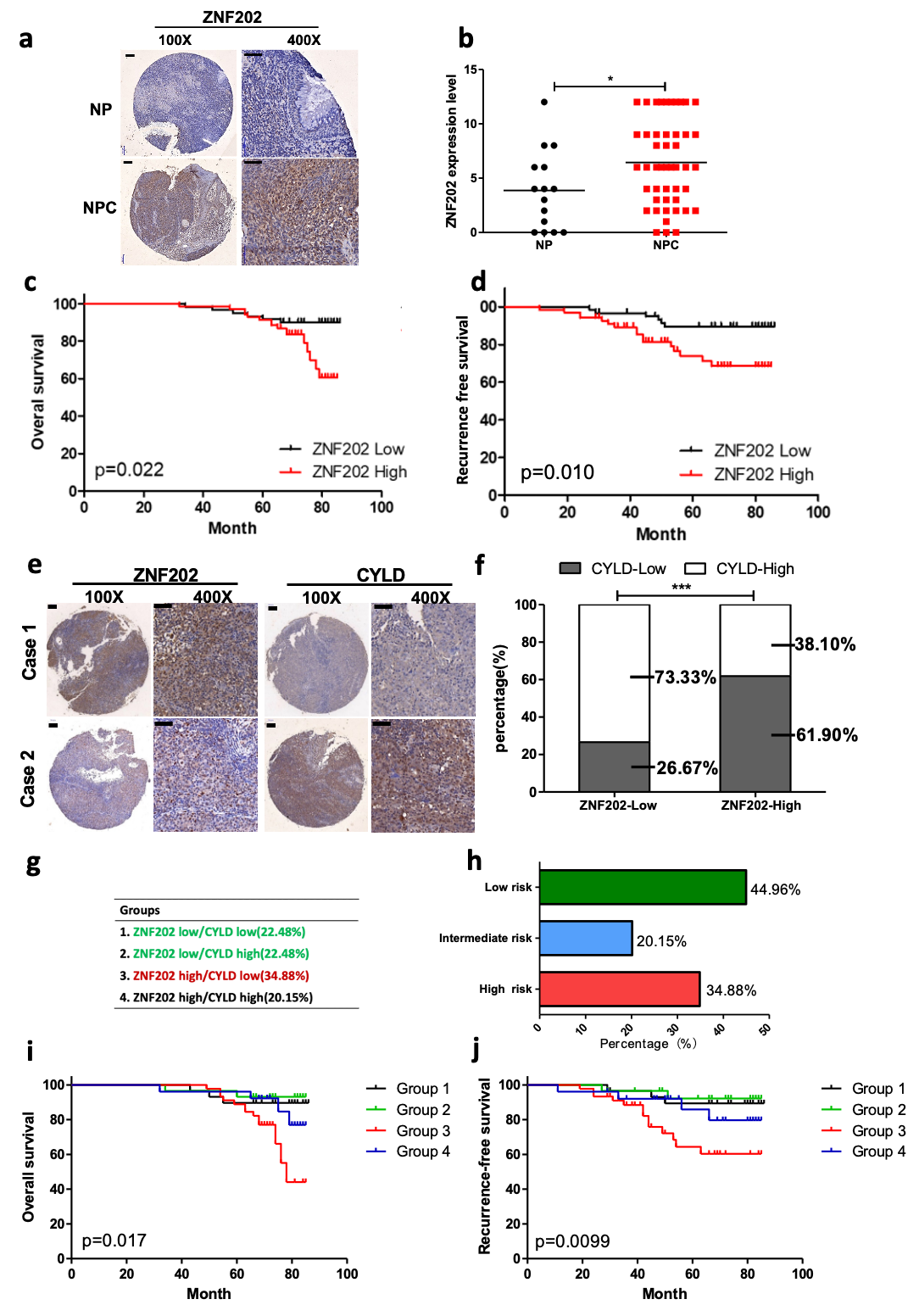
**

**Supplemental Figure 6. High ZNF202 and low CYLD expression is positively associated with poor survival.**

a) Representative IHC staining of ZNF202 expression from an NPC tissue microarray. b) The ZNF202 protein expression level was calculated. c) Overall survival and d) Recurrence-free survival rates of patients were estimated by the Kaplan–Meier method using log-rank test, the median score of ZNF202 was set as cut-off value and divided patients into high- and low-expressing groups. e) Representative IHC staining of ZNF202 and CYLD expression from tissue microarray of nasopharyngeal squamous cell carcinoma patients. f) The CYLD protein expression level was calculated according to ZNF202 expression of nasopharyngeal squamous cell carcinoma patients. High- and low-expressing groups were classified according to median score. g) The percentage of four groups was calculated based on the levels of ZNF202 and CYLD. The median scores of ZNF202 and CYLD were set as the cut-off value and divided patients into high- and low-expressing group. h) Patients were classified into 3 risk groups based on ZNF202 and CYLD levels. i) Overall survival and j) recurrence-free survival rates of patients in four groups were estimated by the Kaplan–Meier method using the log-rank test. Results are plotted as the mean surviving fraction ± SEM of 3 independent experiments.
