# Supplementary material for: CYLD induces high oxidative stress and DNA damage through class I HDACs to promote radiosensitivity in nasopharyngeal carcinoma
Source: Cell Death Dis. 2024 Jan 29;15(1):95. doi: 10.1038/s41419-024-06419-w (PMC10824711; doi:10.1038/s41419-024-06419-w)
Supplement: Supplementary file 3 — Original Data File [file 41419_2024_6419_MOESM3_ESM.docx]

**Uncropped western blots**

**Uncropped western blots for Figure 1**

**
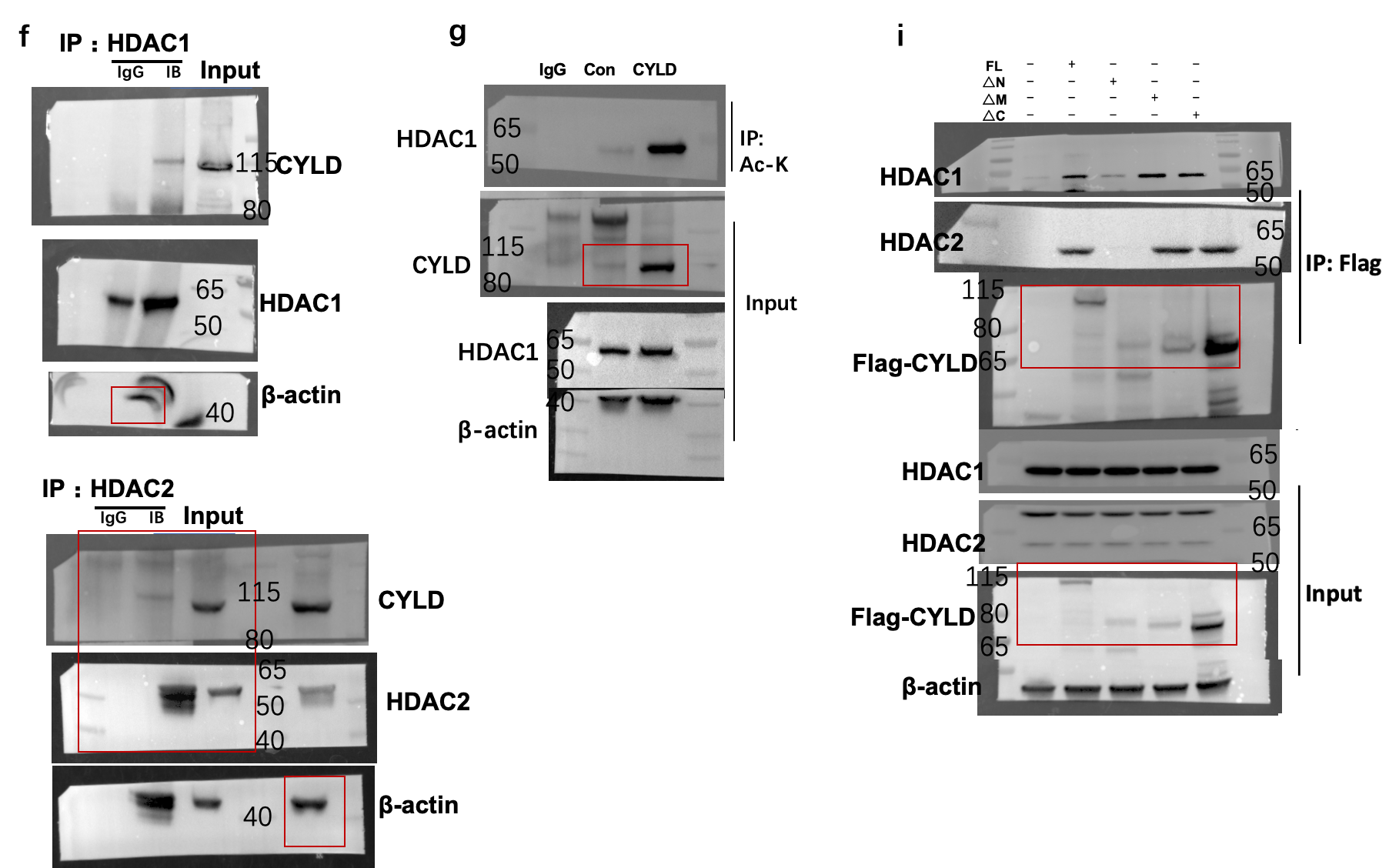
**

**Uncropped western blots for Figure 3**

**
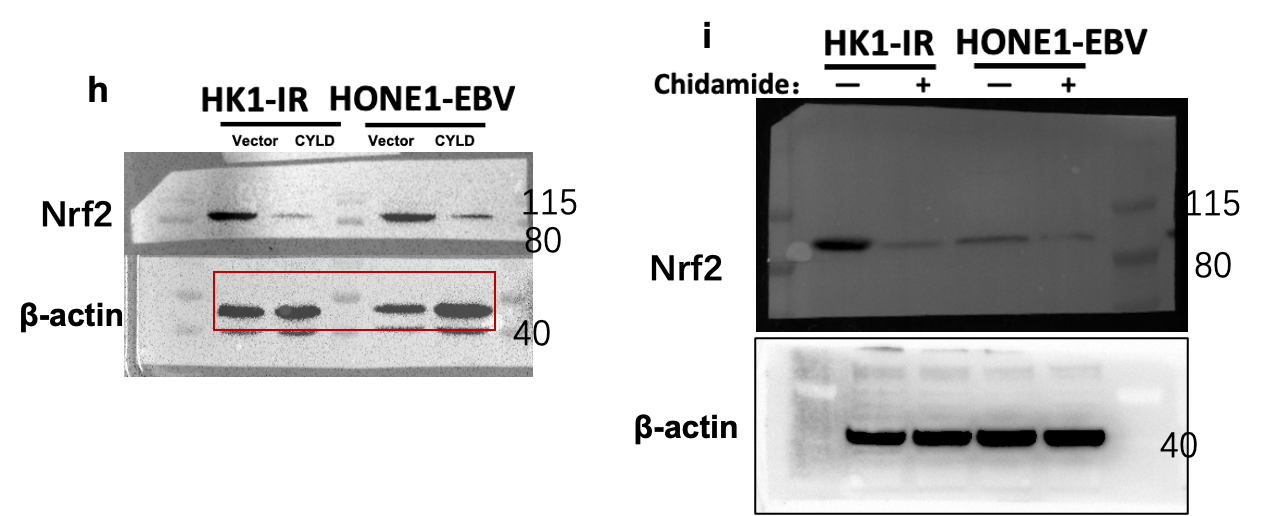
**

**Uncropped western blots for Figure 4**

**
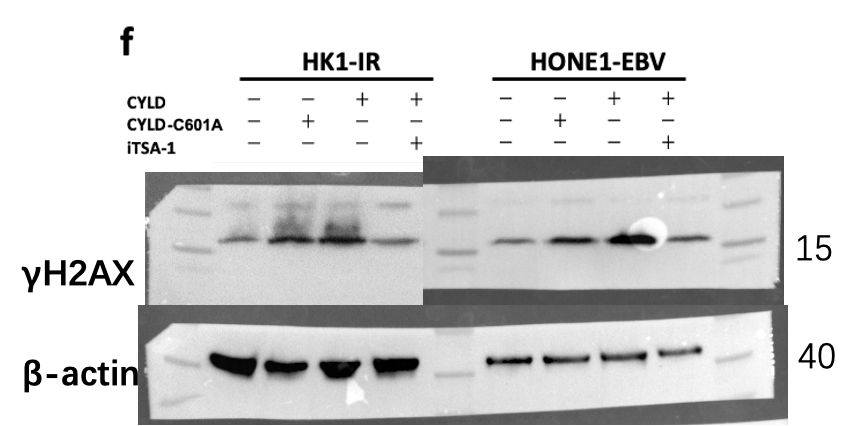
**

**Uncropped western blots for Figure 5**

**
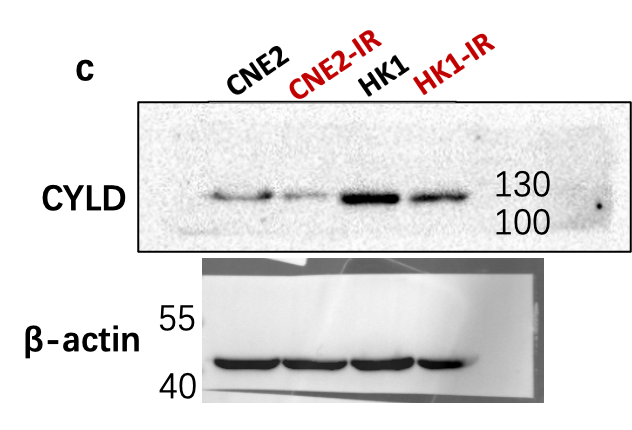
**

**Uncropped western blots for Figure 8**

**
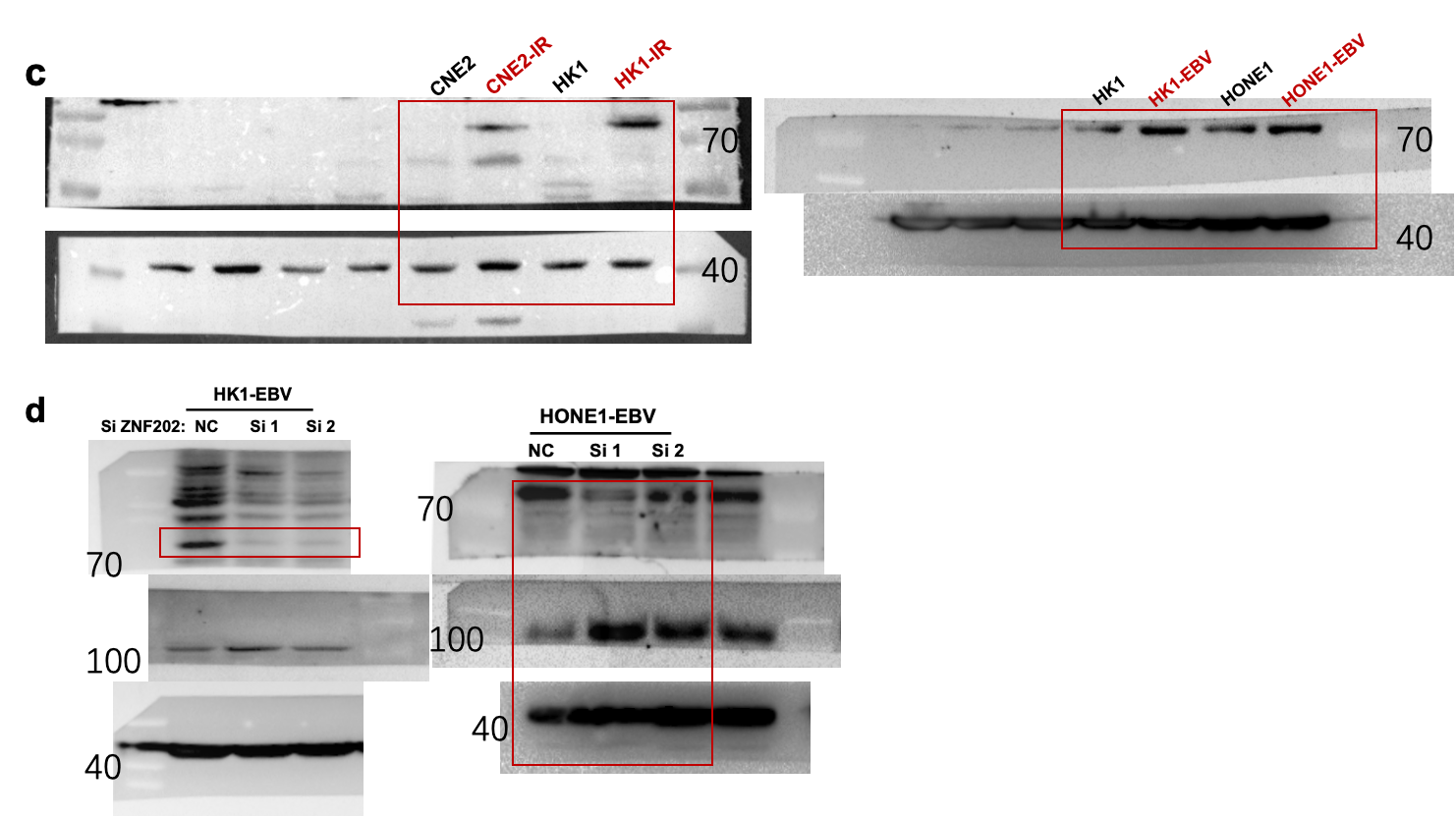
**

**Uncropped western blots for Supplemental Figure 4**

**
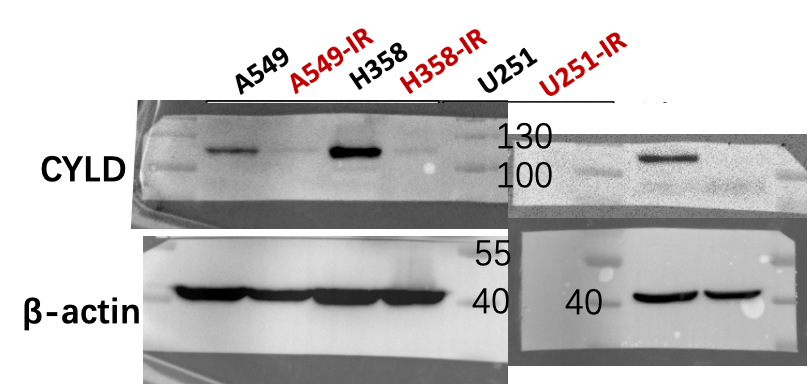
**
